# Dietary lead modulates the mouse intestinal microbiome: Subacute exposure to lead acetate and lead contaminated soil

**DOI:** 10.1016/j.ecoenv.2022.114430

**Published:** 2022-12-20

**Authors:** S. Elizabeth George, Richard Devereux, Joseph James, Yongshan Wan, Gary L. Diamond, Karen D. Bradham, David J. Thomas

**Affiliations:** a U. S. Environmental Protection Agency, Office of Research & Development, Center for Environmental Measurement & Modeling, Gulf Ecosystem Measurement & Modeling Division, Gulf Breeze, FL 32561, United States; b SRC, Inc., North Syracuse, New York 13212, United States; c U. S. Environmental Protection Agency, Office of Research & Development, Center for Environmental Measurement & Modeling, Research Triangle Park, NC 27711, United States; d U. S. Environmental Protection Agency, Office of Research & Development, Center for Computational Toxicology & Exposure, Chemical Characterization & Exposure Division, Research Triangle Park, NC 27711, United States

**Keywords:** Lead, Intestinal microbiome, Mining soil, Bioavailability, Microflora modulation, Predicted functional genes, Mice

## Abstract

The effect of dietary lead on the intestinal microbiome has not been fully elucidated. To determine if there was an association between microflora modulation, predicted functional genes, and Pb exposure, mice were provided diets amended with increasing concentrations of a single lead compound, lead acetate, or a well characterized complex reference soil containing lead, i.e. 6.25–25 mg/kg Pb acetate (PbOAc) or 7.5–30 mg/kg Pb in reference soil SRM 2710a having 0.552 % Pb among other heavy metals such as Cd. Feces and ceca were collected following 9 days of treatment and the microbiome analyzed by 16 S rRNA gene sequencing. Treatment effects on the microbiome were observed in both feces and ceca of mice. Changes in the cecal microbiomes of mice fed Pb as Pb acetate or as a constituent in SRM 2710a were statistically different except for a few exceptions regardless of dietary source. This was accompanied by increased average abundance of functional genes associated with metal resistance, including those related to siderophore synthesis and arsenic and/or mercury detoxification. *Akkermansia*, a common gut bacterium, was the highest ranked species in control microbiomes whereas *Lactobacillus* ranked highest in treated mice. Firmicutes/Bacteroidetes ratios in the ceca of SRM 2710a treated mice increased more than with PbOAc, suggestive of changes in gut microbiome metabolism that promotes obesity. Predicted functional gene average abundance related to carbohydrate, lipid, and/or fatty acid biosynthesis and degradation were greater in the cecal microbiome of SRM 2710a treated mice. Bacilli/Clostridia increased in the ceca of PbOAc treated mice and may be indicative of increased risk of host sepsis. Family Deferribacteraceae also was modulated by PbOAc or SRM 2710a possibly impacting inflammatory response. Understanding the relationship between microbiome composition, predicted functional genes, and Pb concentration, especially in soil, may provide new insights into the utility of various remediation methodologies that minimize dysbiosis and modulate health effects, thus assisting in the selection of an optimal treatment for contaminated sites.

## 1. Introduction

Industrial mining and smelting of lead (Pb) has contaminated soil (Gamiño-Gutiérrez et al., 2013; Kozlov and Zvereva, 2007), sediment (Gillan et al., 2015; Jones et al., 1991; Rowan et al., 1995), and ground and surface water worldwide (Eichler et al., 2014; Moore and Luoma, 1990). Lead concentrations in Pb and zinc (Zn) mining and smelting soils can range from 10^1^ to 10^4^ mg/kg (Xu et al., 2017). Generally, Zn, cadmium (Cd), copper (Cu), chromium Cr), arsenic (As), nickel (Ni), magnesium (Mg), mercury (Hg), and/or manganese (Mn) are co-contaminants (Beattie et al., 2017; Guo et al., 2017; Li et al., 2017b). Lead compounds such as Pb oxide, carbonate, sulfate, and sulfide have been identified as the primary Pb species in soil collected near a Pb smelter no longer in operation where Pb levels ranged from 1100 to 5300 mg/kg (Scheckel and Ryan, 2004). Environmental Pb contamination can cause serious long-term health effects, especially in children who are primarily at risk of Pb exposure through soil and dust contact (Lanphear et al., 1998; Ryan et al., 2004; Schmitt et al., 1979; Shao et al., 2018; Thornton et al., 1990). The integrated exposure uptake biokinetic model (IEUBK; Hogan et al., 1998, Laidlaw et al., 2017; USEPA, 1994) and more recent Stochastic Human Exposure and Dose Simulation (SHEDS) Multimedia IEUBK model characterize the major role of these Pb exposure routes (Zartarian et al., 2017).

Aside from its reproductive, neurological, genetic, hepatic, nephrological, musculoskeletal, and hemopoietic effects, Pb also is toxic to microorganisms, both in their human or murine host and the environment. While the oral of Pb acetate in rats is 4665 mg/kg (U. S. National Library of Medicine, 2022), effects of subacute exposure of dietary Pb on intestine microbiome is not well understood. Upon exposure to Pb, microorganisms may develop protective mechanisms that select for resistant members and can alter intestinal metabolism. For example, changes in the intestinal microbiome of individuals residing near a mining and smelting area have been correlated with exposure to contaminated soil containing an average of 60 mg/kg Pb and other heavy metals (Shao and Zhu, 2020). Mice exposed nine days to heavy metal contaminated smelter soils, containing approximately 2000–3000 mg/kg Pb (prepared at 0.6 % w/w in diet), resulted in modulation of the intestinal flora (George et al., 2022). When compared to unremediated Pb smelter soil, phosphate amendments (triple super phosphate or phosphoric acid) to soil resulted in reduced Pb bioavailability and significant changes in the fecal microbiome when administered in the diet (Bradham et al., 2018; George et al., 2022). Introduced iron rich waste, which can compete for Pb transport across the intestinal barrier (Bressler et al., 2004), or bio-solids, which have the potential to adsorb and immobilize Pb (Brown et al., 2003), to the phosphate enriched soils altered the microbiome profile, relative amounts of lead species in feces, and relative bioavailability. Furthermore, microorganisms adapt and resistant members of the population become more dominant and can cause dysbiosis in humans or mice which impacts physiological processes including metabolism and immune function (Breton et al., 2013b; Gao et al., 2017; Xia et al., 2018a). Resident environmental microbial communities also may shift from sensitive to tolerant or resistant status, potentially effecting the dynamic behavior of Pb in soil (Gillan et al., 2015; Jie et al., 2016; Van Houdt et al., 2009) which can select for microbial Pb resistance genes, thus increasing gene copy number in the environment (Naik and Dubey, 2011; O’Brien et al., 2014). Because Pb resistance mechanisms involve intracellular and extracellular Pb sequestration such as binding to siderophores, exopolysaccharide or metallothionein, or formation of less soluble Pb species, such as Pb phosphate or Pb sulfide, bioaccessibility and bioavailability can be affected. Therefore, while these Pb resistance mechanisms are intended to be protective, they also contribute to Pb transformation.

Lead mining and smelting soils also contain a complex mixture of heavy metals and other elements (George and Wan, 2020), and exposure to different metals other than Pb can confer varying effects. For example, in As oxide treated mice, carbohydrate transport and metabolism were elevated and inorganic ion transport and metabolism and secondary metabolite biosynthesis were depressed which correlated to elevated Firmicutes, Proteobacteria and Tenericutes and decreased Bacteroidetes and TM7 phyla (Guo et al., 2014). In another study, microflora effects were mediated by the addition of ferric chloride (5 mg/L) and resulted in changes to the abundance of several antimicrobial resistance genes. Pb, Cd, Cu, and/or Al also modulated the intestinal flora (Zhai et al., 2017). Verrucomicrobia were reduced by eight weeks of Pb, Cd, Cu and Al treatment; Tenericutes, Cd and Cu treatment; Actinobacteria, Cu and aluminum (Al) treatment; and Proteobacteria, Pb treatment. These studies were conducted on unaged single or mixtures of pure chemicals prepared in the laboratory which informs, but is not directly reflective of aged contaminated environmental soils. Changes in the intestinal microflora have been shown to occur following exposure in the diet of environmentally sourced soils contaminated with Pb and other heavy metals, yet it is not clear how different it is from the exposure to dietary Pb as Pb acetate. Better controlled studies are needed to elucidate the differences.

In this study, mice were fed dietary Pb as Pb acetate (PbOAc) or in a well characterized complex heavy metal laden soil, National Institute of Standards and Technology (NIST) Standard Reference Material (SRM) 2710a. The aims of this study are to 1) determine the effect of subacute dietary Pb exposure of mice on the cecal and fecal microbiome at varying taxon levels; 2) identify microflora similarities and differences between the two treatment groups; 3) link changes in the microbiota to potential health related effects using established indicators and functional analysis; and 4) determine if microbiome modulation correlates with mammalian Pb bioavailability. Understanding the relationship between intestinal microbiome composition, Pb concentration, and influence of complex soils, such as mining soils, may ultimately provide new insights into the intestinal microbiome’s role in Pb transformation. In turn, this may inform the utility of various remediation methods that modulate Pb effects and thus help risk assessors and risk managers in selection of an optimal treatment for a specific contaminated site.

## 2. Material and methods

### 2.1. Test Materials

Lead acetate trihydrate (PbOAC; Sigma-Aldrich, St. Louis, MO) and Standard Reference Material^®^ 2710 (SRM 2710a, National Institute of Standards & Technology, Gaithersburg, MD) were used in this study. SRM 2710a is identified as a Montana I and “Highly Elevated Trace Element Concentrations Soil”. Soil was obtained from a flood plain near Butte, MN, and was dried, ball-milled, and blended with Pb oxide to a final concentration of 0.55 % (National Institute of Standards And Technology, 2018). Metals include As (0.154 %), Cu (0.342 %), iron (Fe; 4.32 %), Pb(0.552 %), Mg (0.734 %) titanium (Ti; 0.311 %), Zn (0.418 %), Cd(12.3 mg/kg), cobalt (Co; 5.99 mg/kg), and Hg (9.88 mg/kg). The majority of particles were sized 1–100 μm (range 0.5–200 μm); other elements analyzed are listed on the Certificate of Analysis.

### 2.2. Mouse exposure

Twenty-eight-day old female C57BL/6 mice (Charles River Laboratories, Raleigh, NC, USA) were acclimated for 12–13 days and then housed in groups of three in metabolic cages (Lab Products, Seaford, DE). Mice were provided a diet ad libitum comprised of PbOAc (12 mice/Pb concentration tested: PbOAc; 25, 12.5 and 6.25 mg/kg Pb;) or SRM 2710a (9 mice/Pb concentration tested: 30, 15 and 7.5 mg/kg Pb;) in powdered AIN-93 G rodent diet (Dyets, Bethlehem, PA). Control mice (9 mice/treatment) received a diet without Pb. Monitoring of diet and water consumption and collection of feces samples from each metabolic cage (Lab Products, Seagrove, DE) was done on Day 2 and Day 9 and followed standard assay procedures. Animals were euthanized with CO_2_ on Day 9 and ceca removed; three ceca were harvested from animals at each PbOAc Pb concentration (3 animals x 3 concentrations Pb), three ceca from each SRM 2710a Pb concentration (3 animals x 3 concentrations Pb), and three ceca from control animals were used for 16 S rRNA analysis. No evidence of significant adverse effects on body weight, food consumption, or water consumption were observed (Table S1). Cecal and fecal samples were stored at − 20 °C. The uptake of Pb into bone, blood, liver, and kidney of mice were measured and reported separately (Bradham et al., 2016). The protocol using mice was reviewed by the Institutional Animal Care and Use Committee of the U. S. Environmental Protection Agency, Research Triangle Park, North Carolina. The studies reported here conformed to the approved protocol that complied with National Institutes of Health guidelines for the care and use of laboratory animals (NIH Publications No. 8023, revised 1978).

### 2.3. 16S rRNA gene sequencing

Fecal and cecal content samples from three cages/treatment were shipped frozen to the DNA Environmental Sample Preparation and Sequencing Facility at Argonne National Laboratory (Lemont, IL). There, DNA was extracted and the V4 region of the 16 S rRNA gene of Bacteria and Archaea was amplified by polymerase chain reaction (PCR) with extracted DNA as template. The amplicons were sequenced on a MiSeq sequencer platform (Illumina, Inc., San Diego, CA) according to procedures used by the Earth Microbiome Project (Thompson et al., 2017).

### 2.4. Data analysis

DNA sequences were processed with *mothur* v.1.43 software using a standard set of commands (Schloss et al., 2009). The mothur cluster.split command was used to construct the distance matrix and sequences having > 97 % nucleotide identities were clustered within an Operational Taxonomic Unit (OTU). The percent recovery of OTUs in a sample was estimated using the equation of Good (Good, 1953). Families and phyla were identified in *mothur* using the SILVA v132 non-redundant 16 S rRNA data base (Quast et al., 2012). Shannon diversity, Margalef richness and Pielou evenness were calculated from OTU data (Clarke et al., 2006).

The 16 S rRNA gene abundance data were fourth root transformed and a Bray-Curtis resemblance matrix and non-metric multidimensional scaling (nMDS) ordination plots generated (Clarke et al., 2006). Cecal and fecal microbial community compositions were analyzed for changes following exposure to different forms and concentrations of Pb with Primer 7 (PRIMER-e, Quest Research Limited, Auckland, New Zealand). Effects within and between treatments were ascertained using PERMANOVA+ and ANOSIM. Contributions of individual OTUs or taxa to dissimilarity between treatments were identified by SIMPER. The significance level of all statistical analyses was set at α = 0.05. Because Pb and other heavy metal concentrations used in this study were relatively low with a shorter duration of exposure than studies in the literature (Breton et al., 2013b; Liu et al., 2014; Xia et al., 2018a; Zhai et al., 2017), *p* values between 0.05 and 0.1 were considered trending significant for differences between treatments.

Functional analysis of the top 20 OTUs accounting for the dissimilarity at the highest dose of PbOAc (25 mg Pb/kg) or SRM 2710 (30 mg Pb/kg) was conducted with the CLC Genomics Workbench 22.0.2, Microbial Genomics Module (QIAGEN, Aarhus, DK). The Infer Functional Profile was used with the PICRUSt2 Multiplication Table (Douglas et al., 2020) and Enzyme Commission number database from Expasy (Bairoch, 2000) (https://enzyme.expasy.org/). The tool identifies the pathway and pathway ID. MetaCyc (metacyc.org; Caspi et al., 2020) and Transform Column-All Ancestors of Entity were used to identify super pathways.

## 3. Results

### 3.1. PbOAc and SRM 2710a alter mouse cecum and feces bacterial community structure

The cecal and fecal 16 S rRNA gene sequence libraries, when processed together in *mothur* and subsampled to 5000 sequences/sample, resulted in a dataset having of 11,202 OTUs across all samples. This dataset was analyzed first for comparisons among sample types. Because a fecal sample had the lowest numbers of sequences, cecal samples alone were subsampled to 41,931 sequences/sample providing a data set with 4556 OTUs. The 16 S rRNA sequences represented 97–99 % of the cecal and fecal microbiomes by the estimate of Good (Good, 1953).

**Fig. 1.**
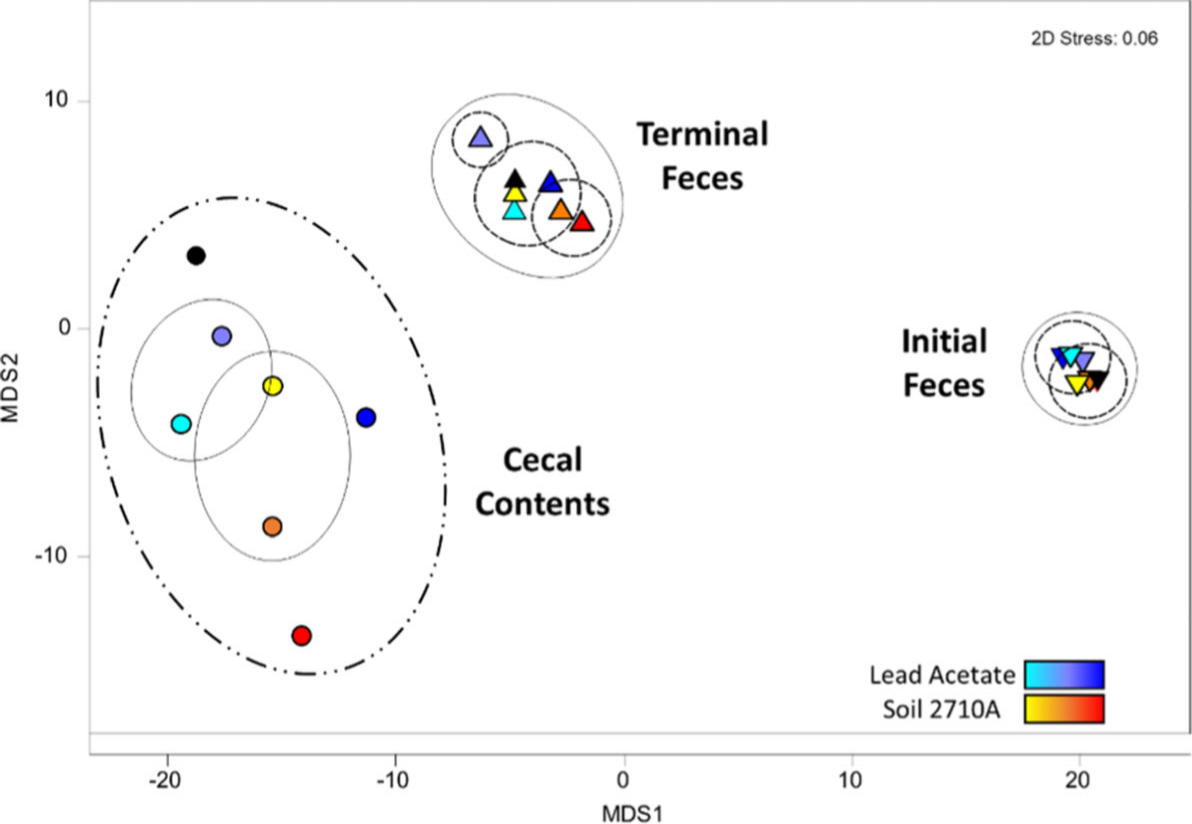
Relationship of OTUs among sample type, Pb amendment, and Pb dose in a metric multidimensional scaling plot. Lines enclose groups based on Bray-Curtis distances: dot-dash, 30; solid gray, 25; dotted 20. Analysis based on 5000 OTU sequences per sample. Circles represent cecal contents on day 9; inverted triangles, fecal contents on Day 2; and triangles, fecal contents on Day 9. Lead acetate dietary doses are represented by aqua, 6.25 mg/kg; purple, 12.5 mg/kg; and navy, 25 mg/kg; Pb SRM 2710a dietary doses are represented by yellow, 7.5 mg/kg; orange, 15 mg/kg; and red, 30 mg/kg. Black fill designates the control with 0 mg/kg Pb.

Cecal and fecal communities differed greatly from each other as shown by separation in a nMDS plot (Fig. 1). In terms of Bray-Curtis distances by treatment and dose level, the initial fecal communities were the most tightly grouped, whereas the cecal communities were most dispersed. Among cecal samples, the control, 25 mg/kg PbOAc treatment, and 30 mg/kg Pb in SRM 2710a treatments differed most from each other with Bray-Curtis dissimilarity scores between 25 and 30. Cecal PbOAc-treatments at 12.5 mg/kg and 6.25 mg/kg dietary Pb, and cecum 15 mg/kg and 7.5 mg/kg dietary Pb in SRM 2710a samples, had Bray-Curtis dissimilarity scores between 20 and 25. The initial and terminal treatment fecal communities grouped with Bray-Curtis distances between 20 and 25. Values for numbers of OTUs, richness, evenness and diversity in ceca were higher with treatment compared to control samples (Table S2). In contrast, Shannon diversity scores between treatment and control samples varied slightly.

### 3.2. Subacute Pb exposure impact on mouse cecal microbiome

Cecal sequences/out/sample were summed into taxonomic groups obtained with SILVA then analyzed by PERMANOVA (Table 1). Significant differences between PbOAc and SRM 2710a treatments were detected across all taxonomic levels except for phylum. When compared with the control, the treatment effect of SRM2710a was significant at taxonomic levels of genus, family, order, and class, and trending significant at the phylum level. In contrast, the treatment effect of PbOAc was significant only at the phylum level.

**Fig. 2.**
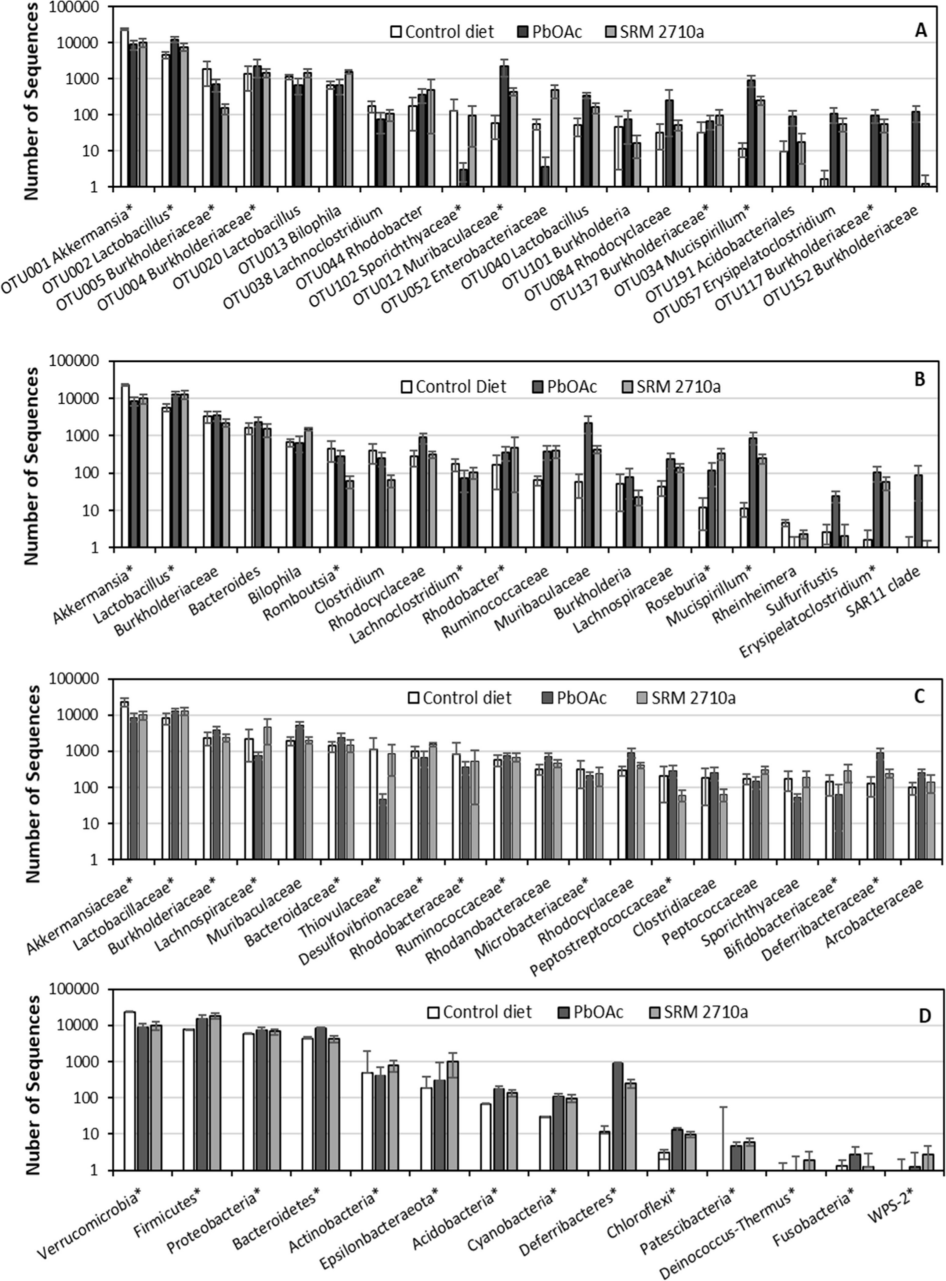
Cecal microbiome contributing to dissimilarity between control diet and treatments classified at taxonomic levels of OTU (A), genus (B), family (C), and phylum (D) selected by SIMPER analysis. The top twenty that contribute most to dissimilarity between PbOAC treatment and control are plotted. Values are mean numbers of sequences across treatment doses with error bars being the standard error of the mean. Control diet taxa are in rank order. Taxa identifications use the lowest taxon having a recognized name; asterisk indicates taxon was among the 20 top contributors to dissimilarity from control in SRM 2710a treatments.

SIMPER provides the contribution of each variable, e.g., an OTU, to the percent similarity/dissimilarity among communities (Clarke et al., 2014). The 84–94 cecal OTUs contributing 70 % similarity within control and treatment microbiomes are provided in Table S4. Twenty OTUs contributing most to dissimilarity between PbOAc treatment and control were selected to demonstrate changes in microbiomes with treatment (Fig. 2A; Table S4). OTU001, identified as *Akkermansia* was the top ranked control OTU and a main contributor to dissimilarity with Pb treatment. *Lactobacillus* was the top ranked OTU for PbOAc treatment, with OTU004 (*Burkholderia*), and OTU012 (Muribaculaceae) among others also ranking higher with PbOAc or SRM 2710a treatment. Five Burkholderiaceae OTUs accounted for near 3 % dissimilarity between control and PbOAc treatment (Fig. 2A). Three of the five *Burkholderia* OTUs had higher ranks in treated microbiomes, whereas Burkholderiaceae OTU005 ranked third in control, had lower ranks in treatments. Most of the top 20 OTUs in common varied similarly with either treatment relative to control, with OTU102 Sporichthyaceae and OTU052 Enterobacteraceae having a lower rank with PbOAc than SRM 2710a treatment.

OTU sequences compiled into *Akkermansia*, *Lactobacillus,* Burkholderiaceae and *Bacteroides*, dominated ceca of control, PbOAc and SRM 2710a microbiomes (Fig. 2B, Table S3). Notably, *Akkermansia* was the top ranked genus among control ceca samples with *Lactobacillus* ranked first in the ceca of treated mice. Genera within Ruminococcaceae, Muribaculaceae, Lachnospiraceae, Mucisprillum, and Erysipelatoclostridium had higher ranks in treated ceca than control (Table S5). *Romboustia* and *Clostridium* ranked lower in SRM 2710a treatments compared to control.

Approximately 90 % of the OTUs were identified at the family level. Rank order of families identified as contributing most to dissimilarity were variable among treatments (Fig. 2 C; Table S6). Compared to control, PbOAc families Akkermansiaceae, Lachnospiraceae, Thiovulaceae, and Sporichthyaceae had lower ranks whereas Muribaculaceae, Rhodocyclaceae, and Deferribacteraceae and Acrobacteraceae had higher ranks. Change in rank order for SRM 2710a families compared to control was limited to lower ranks for Peptostreptococcaceae and Clostridiaceae.

Relative abundances of sequences by class varied greatly. Verrucomicrobiae relative abundance decreased from over 50 % in control to 20–30 % for treatments (Fig. S1). The class *Bacilli* doubled in relative abundance from 15 % in both treatments, as did Clostridia from 0.5 % in SRM 2710a cecal contents. Phyla Verrucomicrobia, Firmicutes, Proteobacteria, and Bacteriodetes sequences were prevalent in the ceca of untreated mice and approximately 3- to 10-fold higher in relative abundance than the remaining phyla (Fig. 2D). Firmicutes was top ranked in treated and Verrucomicrobia was top ranked in control mice; Deferribacteres also ranked higher in the treatments than control. Relative abundance of cecal Bacteroidetes increased in PbOAc treated mice and Firmicutes increased with PbOAc and SRM 2710a treatments. The P(perm) > 0.2 between PbOAc and SRM 2710a phyla is reflective of similar Bacteroidetes and Deferribacteres abundances. Cecal Firmicutes/Bacteroidetes and Bacilli/Clostridia relative abundance ratios are shown in Table 2.

### 3.3. Subacute Pb exposure impact on mouse fecal microbiome

PERMANOVA of OTU data indicated treatments (PbOAc or SRM2710a) were not significantly different from control with *P*(perm) > 0.05 for each dietary amendment though Bray-Curtis similarities for bacterial communities in Day 9 feces differed significantly from feces at the start of the experiment (Table 3). As described above, and seen in Fig. 1, the divergence of fecal communities was not as great as observed in the cecum. Initial control and terminal control samples differed with *P* (perm) = 0.098. However, initial treatment vs. initial control, or terminal treatment vs terminal control, were not significantly different for both treatment types. Nonetheless, significant differences in OTU ranks between PbOAc and SRM 2710a treatments were observed on Day 9 (*P* (perm) = 0.009). Pb dose level had a limited effect on fecal microbiomes when analyzed by PERMANOVA [*P*(perm) ≥ 0.1]. PERMANOVA tests were based on community-wide analyses; any treatment effects that might have occurred within the 9-day experiment will require a finer level of analysis to uncover.

**Fig. 3.**
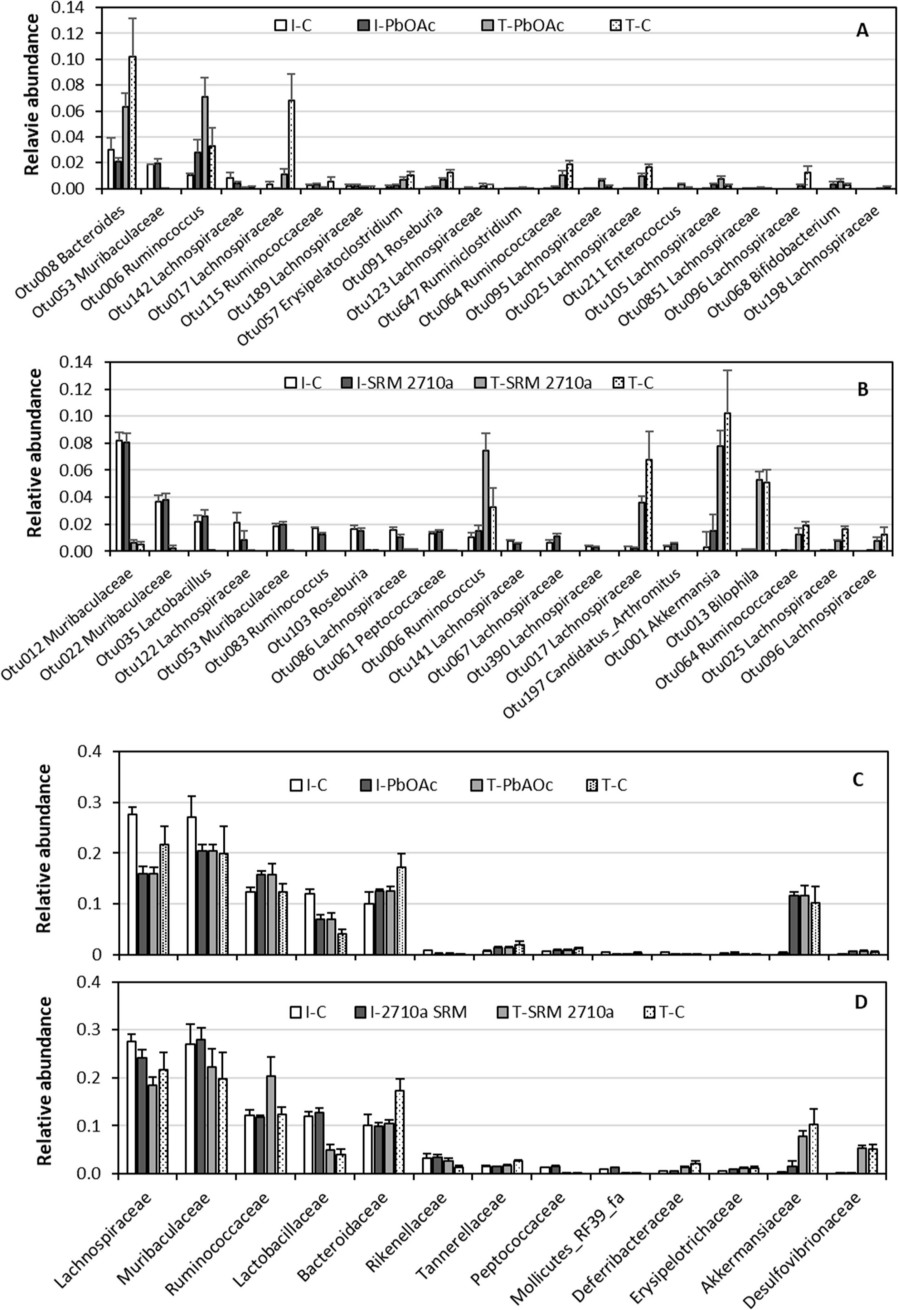
Relative abundance of fecal microbiomes: OTUs in PbOAc (A), OTUs in SRM 2710a (B), families in PbOAc (C), and families in SRM 2710a (D) selected by SIMPER analyses. Families with the most sequences in each treatment were selected for the plots. I=initial Day 2, T = terminal Day 9, C=control without dietary Pb. Nomenclature from SILVA. Values are means with error bars being the standard error of the mean.

OTU 006, identified as *Ruminococcus*, was ranked higher in both Day 9 treatments compared to Day 2 and Day 9 controls (Fig. 3A; Fig. 3B). Ruminococcaceae OTU 064 in treatments ranked higher than Day 2 and lower than Day 9 controls. According to SIMPER analysis, OTUs identified as *Lachnospira* (or within the family Lachnospiraceae) accounted for 10 and 8 of the 20 most important OTUs in PbOAc and SRM 2710a treatments, respectively. The rank of individual *Lachnospira* OTUs varied by treatment with Lachnospiraceae OTU017 being higher than initial but lower than terminal control (Fig. 3A; Fig. 3B).

Approximately 77 % of the fecal microbiome OTUs from all treatments were assigned a family level identification (Fig. 3C; Fig. 3D;). Muribaculaceae, Lachnospiraceae, Ruminococcaceae, Bacteroidaceae, Rikenellaceae, Lactobacillaceae, and Akkermansiaceae were dominant. No community level treatment effects of families across treatments were observed by PERMANOVA. However, Lachnospiraceae and Bacteroidaceae relative abundances were reduced in both treatments, whereas Ruminococcaceae were higher, compared to Day 9 control feces. Responses of additional families were more treatment specific. For example, Lactobacillaceae relative abundance was greater in the PbOAc treatment, but not the SRM 2710a treatment, compared to Day 9 control.

### 3.4. Predicted cecal functional genes

Prediction of functional profiles for the twenty highest cecal OTU contributors to dissimilarity between PbOAc or SRM 2710a and the control revealed fluctuations in metal and antibiotic resistance and metabolism genes (Table 4; Table S7). Siderophore related genes, involved in Pb and metal resistance (Naik and Dubey, 2011; O’Brien et al., 2014), were 10^2^ and 10^1^ times greater than observed in the control for PbOAc and SRM 2710a treated mice, respectively. Genes linked to Hg and As resistance also were elevated in the cecal microbiome from SRM 2710a treated mice. While As genes were more prevalent in the microflora from PbOAc treated mice compared to control, they were four-fold lower than that observed in SRM 2710a treated mice. Average abundance of genes linked to polymyxin, jadomycin, and phenazine compounds also were elevated in both treatments compared to control. In addition, modulation of gene abundance linked to carbohydrate, lipid, and or fatty acid metabolism also was observed.

## 4. Discussion

### 4.1. Environmentally relevant soil and lead acetate differentially effect cecal and fecal microbiome composition

Significant differences in mice intestinal microbiome between treatments of PbOAc and SRM 2710a are indicative of the effects of both Pb bioavailability and Pb speciation in soil matrix. When Pb is introduced to mice as a dietary constituent of a complex soil matrix, relative bioavailability (RBA, compared to PbOAc) decreases (Bradham et al., 2016, 2018; Freeman et al., 1994; Juhasz et al., 2014) and fecal Pb species differ (Bradham et al., 2019; George et al., 2022; Juhasz et al., 2014). Formation of Pb phosphates, whether in the gut or in soil, is beneficial to survival of microorganisms due to their reduced bioaccessibility (George and Wan, 2020). Hydroxyapatite- and organic material-Pb were present in the feces of PbOAc treated mice (Bradham et al., 2019). Lead species present in the SRM 2710a diet included trilead diphosphate, plumbojarosite, and anglesite which also were detected in the feces, suggesting that they did not undergo significant mammalian or microbial metabolism. However, transformation of these three recalcitrant compounds did occur at some level because trilead diphosphate, organic material-Pb, and adsorbed-Pb also were detected. Generally, particles greater than ~100–150 nm have less efficient uptake in the intestines (Desai et al., 1996; Jani et al., 1990) or intestinal epithelial cells (Yao et al., 2015); the majority of particles in SRM 2710a are sized ~1–100 μm (range ~0.5–200 μm). If Pb adsorbed to organic or inorganic SRM 2710a particles as it transited the GI tract, the particles may have been too large to cross the mucosal barrier, making Pb more available to the microflora in the cecum.

In this study, the murine cecal microbiome was differentially affected by PbOAc and SRM 2710a treatment (Table 1). This observation supports a possible link between SRM 2710a related changes in the cecal microflora and reduced Pb bioavailability relative to PbOAc in bone, blood, and kidney. Both PbOAc and SRM 2710a modulate the flora, albeit differently, and the Pb in PbOAc has 100 % RBA compared to an overall 49 % RBA of Pb in SRM 2710a (blood [42 %], liver [60 %], kidney [60 %], bone [34 %]; Bradham et al., 2016). The microflora differences may be due to Pb and co-contaminating metals in the soil. Microbial resistance to Cd and Pb occurs through both similar and different mechanisms. Both Pb and Cd can adsorb to exopolysaccharide, siderophores, or be sequestered intracellularly by binding to metallothionein (Murthy, 2011; Naik et al., 2012; Naz et al., 2005; Ozturk et al., 2010). Both Pb and Cd are removed from cells through efflux mechanisms however the *pbrTRABCD* operon is specific for Pb (Borremans et al., 2001; Hynninen et al., 2009); Cd efflux occurs through a divalent metal Zn/Cd/Pb efflux transporter which can transport other divalent metals (Chen et al., 2015; Leedjarv et al., 2008). Both Cd and Pb cross the intestinal barrier through the divalent metal transport 1 system and may compete for receptor binding (Bressler et al., 2004).

The family Desulfovibrionaceae, which includes the genera *Desulfovibrio* (sulfate reducer; Okabe, 1993) and *Bilophila* (sulfite reducer and linked to inflammation; Feng et al., 2017), had slightly higher relative abundances in the feces, compared to Day 9 controls, and in the ceca of SRM 2710a treated mice. Sulfate and sulfide metabolizing bacteria may have been stimulated by oxidized sulfur in the soil and could be expected to contribute to higher Pb sulfide levels in the feces. Even though anglesite (PbSO_4_) was present in a diet prepared from Pb containing mining soil, the reduced form, Pb sulfide, was not detected in the feces of mice (Bradham et al., 2018) suggesting either that sulfide is scavenged by other oxidants, or the sulfate-reducers are using an alternative metabolism pathway such as fermentation. In a second study, relative concentrations of plumbojarosite (PbFe_6_(SO_4_)_4_(OH)_12_) were similar in both diet and feces of mice treated with SRM 2710a suggesting no sulfate reduction or other transformation of this compound as it transited through the GI tract (Bradham et al., 2019;Karna et al., 2021) and the very low solubility of plumbojarosite (the solubility product or Ksp = 10^−26.2^; Lindsay, 1979). Pb induced perturbations of the intestinal microbiome can result in an increase of Pb tolerant or resistant microorganisms within the community, thus changing microbial balance (An et al., 2018; Zhai et al., 2017). This phenomenon would support reduced relative Pb bioavailability (Bradham et al., 2019) when mice are treated with dietary SRM 2710a compared to PbOAc.

Predicted functional analysis revealed an increase in average gene abundance linked Pb, As, and Hg detoxification (or resistance) in SRM 2710a treated mice. Genes associated with siderophores, which are involved in an extracellular mechanism associated with Pb, As, and other heavy metal resistance (Braud et al., 2009; Gaonkar and Bhosle, 2013) were 7.5-fold more abundant in the ceca of PbOAc treated mice than those that received SRM 2710a or control. Arsenic detoxification genes were 4-fold less abundant in PbOAc treated mice and Hg detoxification genes were rare. These results suggest that Pb, As, and Hg may share several detoxification mechanisms, however Pb availability may be lower in SRM 2710a because it does not induce one of the Pb resistance mechanisms, i.e. siderophore synthesis, to the extent observed in PbOAc treated mice. The co-contaminating metals in SRM 2710a appear to be available as evidenced by modulation of the microbiome and the average abundance of Hg and As detoxification related genes, and may have a cumulative effect on the microflora and compete with Pb for uptake from the intestine, thus reducing Pb RBA.

Average gene abundances related to polymyxin, jadomycin (angucycline class), and phenazine compound resistance were elevated in the cecal microbiome compared to control in both PbOAc and SRM 2710a treated mice. Metal and antibiotic resistance genes can co-occur and concurrent polymyxin and Zn resistance has been reported (Li et al., 2017a). Phenazine and angucycline related compound genes can be plasmid linked (Novakova et al., 2013; Rui et al., 2012) so co-occurrence with metal resistance genes is possible, however generally not reported in the intestinal tract. However, the phenazine compound pyocyanin, when introduced into the intestinal tract, does modulate both the intestinal flora and metabolism (Peng et al., 2022).

The microbiome effects observed in SRM 2710a treated mice may be influenced by carry over DNA contamination from soil microorganisms. SRM 2710a, was radiation sterilized prior to bottling which should eliminate viable soil related microorganisms and spores (National Institute Of Standards And Technology, 2018). However, this process does not necessarily eliminate 16 S amplifiable DNA (Trampuz et al., 2006). It is possible, that soil related DNA confounded elucidation of the cecal and fecal microbiome because one to two percent of DNA administered orally to mice persists after 8 h in the intestinal tract and feces (Schubbert et al., 1997). Therefore, to elucidate soil biotic contributors to the feces and cecum microbiome, the soil could be re-sterilized prior to mixing with diet; identity of soil microbial constituents could be determined by subtraction of sterile soil flora. However, a second sterilization could modify the chemical constituents, thus disrupting the experiment design. Recreating the complex chemical components in a sterile soil of like physical parameters is possible, however the interactions between organic materials and chemicals might be lost, thereby influencing bioavailability and the associated microorganisms. Microflora analyses from reconstructed metal contaminated soils with varying incubation periods (7 days to 18 months), have been performed with success, however many environmentally contaminated soils have been equilibrating for over 100 years and the shortened timeframe may impact the results (Kuppusamy et al., 2016; Liu et al., 2018; Sobolev and Begonia, 2008; Xu et al., 2019; Xu et al., 2018). Other studies have examined the microbiome from sediments adjacent to the contaminated area (Gillan et al., 2015, Roosa et al., 2014a, Roosa et al., 2014b); this approach also introduces multiple variables. Therefore, in this study, the best available control for the SRM 2017a amended diet, i.e. diet alone, was used to discern modulations in the microbiome, recognizing that DNA bound to soil particles could influence results. Soil-specific changes might be inferred when taxon relative abundance differs compared to no change between control and PbOAc treatment. This occurs with OTU005 Burkholderiaceae and OTU013; *Bilophia*, *Rombustsia*, and *Clostridium*; family Desulfovibrionaceae, and class Clostridia in ceca, and Ruminococceae in the feces. Reasons for the changes are not clear.

### 4.2. Comparative microbiome modulation by dietary and drinking water lead exposure

In a recent mouse study, an extended treatment regime (15 weeks, 0.1 mg/l PbOAc in drinking water) altered both cecal and fecal microbiomes (Xia et al., 2018a). *Parabacteroides* increased significantly and *Dehalobacterium*, among other taxa, decreased in the cecum. Fecal relative abundance of the dominant phyla Firmicutes and Bacteroidetes also changed consistent with Zhai et al. (2017) who observed an increase in Bacteroidetes and decrease in Firmicutes after 8 weeks treatment with 1.83 mg/L PbOAc. Decreases in fecal *Bacteroides* and the family Bacteroidaceae were observed in the current study after just 9 days (Fig. 3C). Fecal Lactobacillaceae relative abundance was increased after 8 weeks of treatment (Xia et al., 2018b) as observed in our study with PbOAc, though not with SRM 2710a (Fig. 3C; 3D). However, in this and other studies *Lactobacillus* species relative abundances were increased for cecal and fecal microbiomes exposed to Pb (Fig. 2B; Breton et al., 2013b, Zhai et al., 2017). Lactobacilli are common in probiotics, reduce inflammation, and bind Pb making it less bioavailable (Arun et al., 2021; Liu et al., 2021). The increased Lactobacilliaceace relative abundances for both ceca and feces exposed to Pb was the only change in common between the microbiomes observed among the most abundant taxa in our study. Relative abundance of family Ruminococcacea sequences in ceca of our study and that of Breton et al. (2013b) was unchanged in contrast to decreased abundance noted by Zhai et al. (2017). *Akkermansia* are considered to limit inflammation; lower relative abundances upon Pb exposure were observed in the current study as has been previously reported (Zhai et al., 2017). Other changes common to our and other studies include increases in Erysipelotrichaceae, *Bukholderia*, and *Desulfovibrio* (Li et al., 2021; Wu et al., 2016).

Perturbations to the communities in some cases remained evident when numbers of sequences were compiled into higher taxonomic levels, likely reflecting profound changes in the microbiomes. With OTUs aggregated at the phylum level, 8 and 15 weeks of PbOAc treatment in previous drinking water studies caused an increase in the relative abundance of Bacteroidetes accompanied by a decrease in Firmicutes in ceca and feces, with Bacteroidetes most prevalent (Xia et al., 2018a; George et al., 2022; Zhai et al., 2017). Further, 8 weeks treatment with 100 mg/L Pb chloride in drinking water decreased the relative abundance of *Bacteroides* in the cecum and to a lesser extent in the feces whereas cecal and fecal *Lactobacillus* trended positive (Breton et al., 2013b). However, no treatment effect was observed for phyla Firmicutes and Bacteroidetes; Firmicutes was dominant in the cecum and comprised 97.8 % and 96.24 % of the microflora in control and treated animals, respectively (Breton et al., 2013b). The fecal microbiome was dominated by Firmicutes in control (64.7 %) and following 8 weeks of Pb chloride treatment (63.85 %). In the current study, relative abundances of cecal Bacteroidetes and Firmicutes increased in PbOAc treated mice, and relative abundances of Firmicutes increased with SRM 2710a treatment (Fig. 2D). All the above studies used mice of approximately the same age, however different Pb sources, concentrations and preparations, and mouse lines, exposure times, sexes or diets were used and may have contributed to the different effects on the microbiome.

### 4.3. Microbial physiological and health indicators associated with dietary lead exposure

**Fig. 4.**
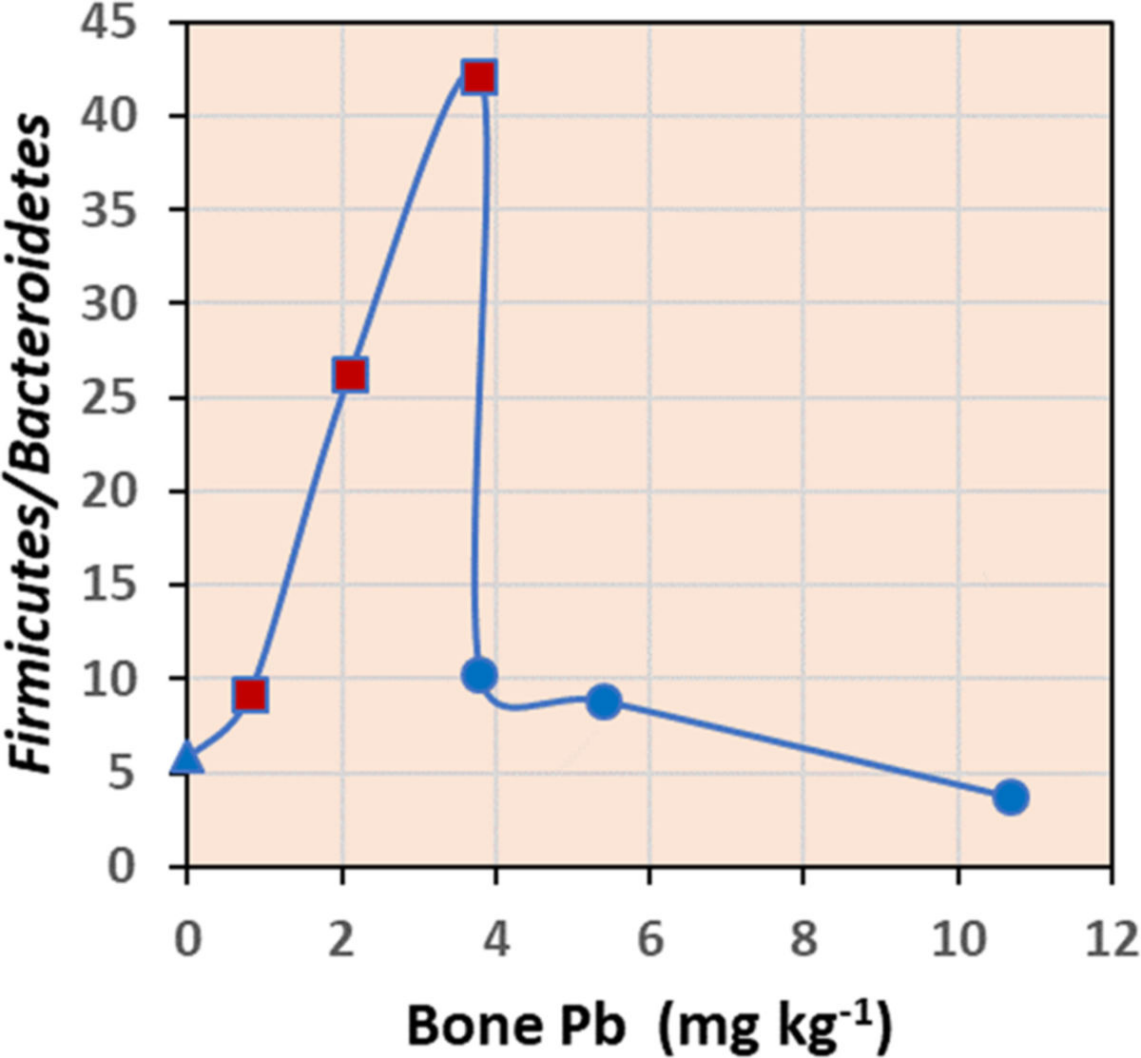
Changes of Firmicutes/Bacteroidetes relative abundance ratio with mouse bone Pb concentration. Bone Pb data were from Bradham et al. (2016). Blue triangle reprents the control, red squares SRM 2710a treatment, and blue circles PbOAc treatment.

Lead exposure alters the mouse intestinal microbiome which in turn can impact gut carbohydrate, energy, and nitrogen metabolism, cause oxidative stress and inflammation, and affect permeability (Gao et al., 2017; Xia et al., 2018b). It is well established that the intestinal microbiome plays an important role in host physiology and metabolism. One indicator, an increase in the Firmicutes/Bacteroidetes relative abundance ratio, has been associated with reduced lipid and carbohydrate metabolism, obesity, high fat diet (Ley et al., 2005; Turnbaugh et al., 2008), and aging (Mariat et al., 2009). The magnitude of changes in the relative abundances of cecal Firmicutes and Bacteroidetes observed in this study were less than the two-fold that previously reported for mice exposed to Pb (Xia et al., 2018a). However, the ratios did vary and were greater than control for most of the treatments (Table 2). Generally, cumulative average abundance of genes associated with carbohydrate, lipid, and fatty acid biosynthesis and degradation were more pronounced in the ceca of SRM 2710a treated mice compared to PbOAc treated or control (Table 4, Table S7). Also, the ratio, when plotted against bone Pb concentration (Fig. 4), exhibited a bi-phase phenomenon, whereby the ratio increased with increasing bone Pb for SRM2710a. In contrast, further increase in bone Pb with PbOAc instigated significant decrease in the ratio, suggesting that exposure to bioavailable Pb is not the sole cause of the change of the ratio. PbOAc selected for both Bacteroidetes and Firmicutes whereas components in SRM 2710a cumulatively selected for Firmicutes. SRM 2710a related toxicants had less impact on Bacteroidetes, possibly due to chemical or physical interaction, causing less toxicity, and remained at control levels. SRM 2710a related ratios correlated with increasing dose, and were comparatively higher than PbOAc related ratios. The SRM2710a co-contaminant Cd may influence the ratio. Three-week exposure with cadmium chloride (20 and 200 mg/L) in drinking water reduced Bacteroidetes relative abundance thus increasing the Firmicutes/Bacteroidetes; short-chain fatty acids synthesis was suppressed (Liu et al., 2014). Additionally, reduction in Firmicutes/Bacteroidetes ratio at the highest PbOAc dose may reflect toxicity within the Bacteroidetes relative to the Firmicutes. Fecal Firmicutes/Bacteroidetes ratios did not correlate with PbOAc and SRM 2710a treatments, so while feces is a more available sample type, it may not be a good indicator of host responses related to Firmicutes and Bacteroidetes.

Order Bacilli/Clostridia relative abundance ratios also have been identified with health status. A reduced Bacilli to Clostridia ratio is indicative of an elevated inflammatory response (Lee et al., 2020; Pearson-Leary et al., 2020), whereas increase in certain Bacilli has been associated with sepsis (Kullberg et al., 2021). Relative abundance of cecal Bacilli increased with both PbOAc and SRM 2710a treatment however the corresponding increase in Clostridia following SRM 2710a treatment contributed a slight effect on the Bacilli/Clostridia relative abundance ratio (Table 2; Fig. S1). A PbOAc treatment effect on the ratio was observed, however increasing dose resulted in a ratio decrease suggesting Bacilli toxicity or a more selective environment for the Clostridia. While this finding could be indicative of sepsis and an elevated inflammatory response, it is only a supposition.

A previous study conducted with this same mouse model found that dietary Pb source (PbOAc or SRM 2710a) was a significant variable in a regression model that explained variance in food efficiency (Bradham et al., 2019). A shift in the gut microbiome serves as a possible explanation for this observation, given the association between Firmicutes and Bacteroidetes abundances and carbohydrate and lipid metabolism. In PbOAc treated mice, Deferribacteraceae, which includes the genus *Mucispirillum* (linked to intestinal inflammatory conditions; Herp et al., 2021) trended positive. Chronic (4–8 weeks) Pb chloride treatment (100 and 500 mg/l) also changed the inflammatory status of duodenum and colon, observed by down-regulation of host mRNA (*Il1b*, *Tnf*, *Ifng*, *Tgfb*, and *Il-10* genes; (Breton et al., 2013a). These observations support a more in depth study on the cecal microbiome metagenome from PbOAc and SRM 2710a treated mice to better understand functional effects.

## 5. Conclusion

Lead acetate or a complex metal laden soil (Pb species predominantly anglesite, plumbojarosite, and hydroxyapatite-associated Pb) was added to the diet of mice to determine the effect on the fecal and cecal microbiome. The result reveals that 1) the treatments differentially modulated the cecal and fecal microbiome; 2) both similarities and differences in the microbiome were observed when aggregated at varying taxon levels; 3) the Firmicutes/Bacteroidetes relative abundance ratio was higher in SRM 2710a treated mice suggesting reduced lipid and carbohydrate metabolism compared to PbOAc; 4) carbohydrate, lipid, and/or fatty acid biosynthetic and degradative pathways, as well as detoxification mechanisms, were modulated with dose, and 5) effects observed did not directly correlate to the pure lead compound suggesting that co-contaminants, such as other metals, were available and may have influenced the results. Although it cannot be confirmed that the differential effect derives solely from the forms of Pb administered in the diet, the Pb dose dependency of the changes suggests the possibility of a connection between the microbiome, average gene abundance related to detoxification and metabolism pathways, and lower bioavailability of Pb in the mining soil. Further research is needed to explore relationships between Pb, co-contaminating elements, and soil induced changes in the gut microbiome. Understanding this relationship may provide new insights into the utility of various remediation methods that modulate the effects and reduce bioavailability. This information may help risk assessors and risk managers in their selection of an optimal treatment for a specific contaminated site.

## Supplementary Material

Supplementary Info.

## Figures and Tables

**Table 1:** PERMANOVA of cecal communities over hierarchical taxonomy. Sequences OTU^−1^ sample^−1^ were summed to taxonomic ranks for PERMANOVA with Monte Carlo tests P(MC). C = control. Statistical significance of *P*(perm) indicated as follows: * * p *<* 0.01, and * p *<* 0.05.

Taxonomic level	Treatment comparison	t	*P* (perm)	Unique perms	*P* (MC)

OTU	PbOAc x C	1.20	0.074	220	0.220
	SRM 2710a x C	1.21	0.063	220	0.182
	PbOAc x SRM 2710a	1.33	0.003 * *	8070	0.052
Genus	PbOAc x C	1.30	0.108	220	0.238
	SRM 2710a x C	1.57	0.055	220	0.138
	PbOAc x SRM 2710a	1.87	0.006 * *	8130	0.043
Family	PbOAc x C	1.18	0.194	220	0.312
	SRM 2710a x C	1.55	0.045 *	220	0.151
	PbOAc x SRM 2710a	2.01	0.002 * *	8130	0.029
Order	PbOAc x C	1.57	0.063	220	0.141
	SRM 2710a x C	1.70	0.032 *	220	0.117
	PbOAc x SRM 2710a	2.00	0.005 * *	8160	0.034
Class	PbOAc x C	1.99	0.059	220	0.079
	SRM 2710a x C	1.89	0.022 *	220	0.091
	PbOAc x SRM 2710a	1.64	0.044 *	8110	0.108
Phylum	PbOAc x C	2.90	0.043 *	220	0.042
	SRM 2710a x C	2.03	0.063	220	0.099
	PbOAc x SRM 2710a	1.19	0.286	8124	0.306

**Table 2:** Firmicutes/Bacteroidetes (F/B) and Bacilli/Clostridia (B/C) ratios in mice ceca. Values are mean (standard error) by treatment and Pb dose (mg/kg).

Treatment	Pb	F/B		B/C	

Control	0	5.84	(0.435)	3.47	(1.39)
PbOAc	6.25	10.2	(0.970)	12.6	(7.38)
	12.5	8.84	(1.20)	9.11	(3.01)
	25	3.71	(0.402)	3.68	(1.02)
SRM 2710a	7.5	9.18	(0.620)	6.51	(1.58)
	15	26.2	(5.50)	2.50	(1.23)
	30	42.1	(11.5)	5.17	(1.56)

**Table 3:** PERMANOVA of feces communities at OTU level for the two dietary treatments: C=control; I=initial feces (Day 2); T = terminal feces (Day 9). For each comparison the degrees of freedom (df), t value (t), *p* values [*P*(perm)], the possible number of unique permutations, and significance level of Monte Carlo tests [*P*(MC)] are provided. Statistical significance of *P*(perm) indicated as follows: * ** *p* < 0.001, * * *p* < 0.01, and * *p* < 0.05.

Treatment type	Treatment comparison	df	t	*P* (perm)	Unique permutations	*P* (MC)

PbOAc	I x I-C	12	1.08	0.138	364	0.309
	I x T	21	3.87	< 0.001 * **	9880	< 0.001
	I x T-C	12	2.77	0.003 * *	364	< 0.001
	T x I-C	13	2.73	0.002 * *	454	< 0.001
	T x T-C	13	1.047	0.274	455	0.359
SRM 2710a	I x I-C	10	1.01	0.416	220	0.429
	I x T	16	3.79	< 0.001 * **	8160	< 0.001
	I x T-C	10	2.96	0.005 * *	220	< 0.001
	T x I-C	10	2.86	0.005 * *	220	< 0.001
	T x T-C	10	1.04	0.268	220	0.380
	T-PbOAc x T-SRM 2710a	19	1.36	0.009 * *	9701	0.044
	I-C x T-C	12	2.61	0.098	10	0.010

**Table 4:** Predicted functional genes from the cecal microbiome of PbOAc and SRM 2710a treated mice compared to the cecal microbiome from animals that received no Pb or other metals. The 16 S sequences from the top 20 OTUs contributing most to dissimilarity at the highest dose of PbOAc (25 mg/kg) and SRM 2710a (30 mg/kg) were analyzed using the CLC Genomics Workbench (22.0.2; Microbial Genomics Module) as described. MetaCyc (metacyc.org) (Caspi et al., 2020) and Transform Column-All Ancestors of Entity were used to identify super pathways.

Predicted Function	PbOAc	SRM 2710a	Control
			
	Average Gene Abundance	Average Gene Abundance	Average Gene Abundance

**Metal Resistance**			
Siderophore	1082	144	7
Arsenic	52	217	0
Mercury	1	431	14
**Antibiotic Resistance**			
vancomycin	19,984	13,963	22,588
vancomycin	11,792	7333	11,545
polymyxin	310	927	38
jadomycin	58,181	89,271	31,767
phenazine compounds	1649	2931	578
tetracenomycin C	176	465	756
**Biosynthesis**			
Carbohydrates	219,846	242,655	208,515
Lipid	151,886	176,104	106,770
**Degradation**			
Carbohydrates	935,609	1668,098	895,936
Fatty Acid & Lipid	84,878	113,694	50,863

## Data Availability

Published article data is available at data.gov. Names of data files are included in the Acknowledgements section. Additional supplemental data (OTU Counts of Ceca and Feces; Silva Taxonomy OTU 1 - OTU 225346) can be found at data.gov.
