# Supplementary material for: Dietary lead modulates the mouse intestinal microbiome: Subacute exposure to lead acetate and lead contaminated soil
Source: Ecotoxicol Environ Saf. Author manuscript; Available in PMC 2024 Jan 1. (PMC10181873; doi:10.1016/j.ecoenv.2022.114430)
Supplement: Supplementary Info. [file NIHMS1855915-supplement-Supplementary_Info_.docx]

Supplemental Information

**Dietary lead modulates mouse intestinal microbiome:**

**Subacute exposure to lead acetate and lead contaminated soil**

S. Elizabeth George^a*^, Richard Devereux^a^, Joseph James^a^, Yongshan Wan^a^, Gary L. Diamond^b^, Karen D. Bradham^c^, and David J. Thomas^d^

^a^U. S. Environmental Protection Agency, Office of Research & Development

Center for Environmental Measurement & Modeling, Gulf Ecosystem Measurement & Modeling Division, Gulf Breeze, FL 32561, United States of America

^b^SRC, Inc., North Syracuse, New York 13212, United States of America

^c^U. S. Environmental Protection Agency, Office of Research & Development

Center for Environmental Measurement & Modeling, Research Triangle Park, North Carolina 27711, United States of America

^d^U. S. Environmental Protection Agency, Office of Research & Development

Center for Computational Toxicology & Exposure, Chemical Characterization & Exposure Division, Research Triangle Park, NC 27711, United States of America

^*^Corresponding Author:

Gulf Ecosystem Measurement & Modeling Division

U. S. Environmental Protection Agency

1 Sabine Island Drive

Gulf Breeze, FL 32561

U. S. A.

George.elizabeth@epa.gov

Table S1. Cumulative cage weights of mice (3 mice/cage) following 9 days of dietary treatment with lead acetate (PbOAc), SRM 2710a, and control diets.

| Treatment | Diet Amendment | Cage | Initial cumulative | Final cumulative |
| --- | --- | --- | --- | --- |
|  |  |  | cage wt (g) | cage wt (g) |
| PbOAc |  | 1 | 51.7 | 53.5 |
|  | 25 ppm Pb | 2 | 50.9 | 53.6 |
|  |  | 3 | 51.9 | 53.1 |
|  |  | 4 | 53.6 | 55.2 |
|  |  | 1 | 51.6 | 51.6 |
|  | 12.5 ppm Pb | 2 | 50.3 | 51.1 |
|  |  | 3 | 50.6 | 51.9 |
|  |  | 4 | 51.1 | 54.9 |
|  |  | 1 | 53.5 | 55.4 |
|  | 6.25 ppm Pb | 2 | 50.9 | 53.2 |
|  |  | 3 | 55.0 | 56.2 |
|  |  | 4 | 52.3 | 52.7 |
| 2710a |  | 1 | 52.4 | 53.5 |
|  | 30 ppm Pb | 2 | 51.8 | 54.8 |
|  |  | 3 | 50.9 | 53.6 |
|  |  | 1 | 52.6 | 55.3 |
|  | 15 ppm Pb | 2 | ` | 53.8 |
|  |  | 3 | 52.5 | 54.1 |
|  |  | 1 | 51.7 | 54.6 |
|  | 7.5 ppm Pb | 2 | 55.4 | 56.9 |
|  |  | 3 | 49.3 | 50.0 |
| Control |  | 1 | 49.5 | 53.2 |
|  | 0 ppm Pb | 2 | 51.9 | 55.5 |
|  |  | 3 | 48.4 | 51.8 |

Table S2. Effects of diet amendments on microbiome diversity. Treatments are averaged for (SE) for control and all dose levels (n, sequenced samples) by amendment. S, number of OTUs; Margalef’s richness; Pielou’s evenness; Shannon diversity index; Simpson diversity index^1^. Analysis based on 5,000 sequences per sample. S and indices determined with Primer 7.

| Sample (n) | Amend. | S | Richness | Evenness | Simpson | Shannon |
| --- | --- | --- | --- | --- | --- | --- |
| Cecum (3) | none | 116.7 (6.39) | 13.6 (0.75) | 0.43 (0.028) | 2.0 (0.15) | 0.68 (0.031) |
| I. feces (3) | none | 317.0 (13.01) | 37.1 (1.53) | 0.73 (0.010) | 4.2 (0.07) | 0.96 (0.004) |
| T. feces (3) | none | 185.0 (11.14) | 21.6 (1.31) | 0.65 (0.022) | 3.4 (0.16) | 0.92 (0.018) |
| Cecum (9) | PbOAc | 126.9 (7.98) | 14.8 (0.94) | 0.52 (0.029) | 2.5 (0.17) | 0.80 (0.035) |
| I. feces (11) | PbOAc | 301.5 (7.93) | 35.3 (0.93) | 0.73 (0.005) | 4.2 (0.03) | 0.97 (0.002) |
| T. feces (12) | PbOAc | 173.5 (8.57) | 20.3 (1.01) | 0.65 (0.011) | 3.3 (0.09) | 0.92 (0.007) |
| Cecum (3) | SRM 2710a | 140.0 (10.68) | 16.3 (1.25) | 0.48 (0.044) | 2.4 (0.25) | 0.74 (0.060) |
| I. feces (9) | SRM 2710a | 314.9 (6.12) | 36.9 (0.72) | 0.72 (0.006) | 4.1 (0.04) | 0.96 (0.003) |
| T. feces (9) | SRM 2710a | 192.4 (7.79) | 22.5 (0.91) | 0.65 (0.015) | 3.4 (0.10 | 0.91 (0.011) |

Table S3. Average sequences per family per cecum sample in control and treated microbiomes. Each sample contained 41,931 sequences. Average similarity across all doses within a treatment, family abundance, average percent similarity a family contributes to within group similarity, average similarity over the standard deviation, and percent contributions to similarity. Average abundance is 4th root transformation of standardized sequences in a family.

| *Control* | | |  |  |
| --- | --- | --- | --- | --- |
| Average similarity: 76.79 |  |  |  |  |
| Family | Average Abundance | Average Similarity | Contribution % | Cumulative % |
| Akkermansiaceae | 2.72 | 4.97 | 6.47 | 6.47 |
| Lactobacillaceae | 1.91 | 3.34 | 4.34 | 10.82 |
| Burkholderiaceae | 1.67 | 2.76 | 3.59 | 14.41 |
| Muribaculaceae | 1.52 | 2.75 | 3.58 | 17.99 |
| Bacteroidaceae | 1.38 | 2.28 | 2.97 | 20.96 |
| Desulfovibrionaceae | 1.11 | 1.91 | 2.48 | 23.45 |
| Ruminococcaceae | 1.04 | 1.86 | 2.42 | 25.87 |
| Lachnospiraceae | 1.07 | 1.74 | 2.27 | 28.14 |
| Chitinophagaceae | 0.92 | 1.69 | 2.21 | 30.35 |
| Rhodanobacteraceae | 0.91 | 1.59 | 2.07 | 32.41 |
| Peptostreptococcaceae | 0.97 | 1.55 | 2.01 | 34.43 |
| Rhodocyclaceae | 0.91 | 1.5 | 1.95 | 36.38 |
| Peptococcaceae | 0.84 | 1.48 | 1.93 | 38.31 |
| Clostridiaceae_1 | 0.91 | 1.34 | 1.74 | 40.05 |
| Pseudomonadaceae | 0.75 | 1.27 | 1.65 | 41.7 |
| Sphingomonadaceae | 0.68 | 1.23 | 1.6 | 43.3 |
| Microbacteriaceae | 0.82 | 1.22 | 1.59 | 44.89 |
| Arcobacteraceae | 0.68 | 1.13 | 1.47 | 46.36 |
| Rhizobiales_Incertae_Sedis | 0.6 | 1.09 | 1.42 | 47.77 |
| Thiovulaceae | 0.65 | 1.09 | 1.41 | 49.19 |
| Chthoniobacteraceae | 0.62 | 1.08 | 1.41 | 50.6 |
| Nitrospiraceae | 0.61 | 1.07 | 1.4 | 51.99 |
| uncultured | 0.59 | 1.06 | 1.38 | 53.37 |
| Enterobacteriaceae | 0.6 | 1.03 | 1.34 | 54.71 |
| Sporichthyaceae | 0.7 | 1.02 | 1.32 | 56.03 |
| Methylophilaceae | 0.56 | 0.94 | 1.22 | 57.25 |
| Spirosomaceae | 0.51 | 0.92 | 1.2 | 58.45 |
| Steroidobacteraceae | 0.53 | 0.9 | 1.17 | 59.62 |
| Micrococcaceae | 0.47 | 0.83 | 1.08 | 60.7 |
| Saprospiraceae | 0.48 | 0.79 | 1.03 | 61.73 |
| Methylomonaceae | 0.46 | 0.79 | 1.02 | 62.75 |
| Rhodobacteraceae | 0.65 | 0.76 | 0.99 | 63.74 |
| Planococcaceae | 0.45 | 0.74 | 0.97 | 64.71 |
| Cyanobiaceae | 0.43 | 0.72 | 0.93 | 65.64 |
| Ilumatobacteraceae | 0.41 | 0.72 | 0.93 | 66.57 |
| Moraxellaceae | 0.44 | 0.71 | 0.92 | 67.5 |
| Beijerinckiaceae | 0.42 | 0.69 | 0.9 | 68.4 |
| Oxyphotobacteria_unclassified | 0.38 | 0.69 | 0.9 | 69.3 |
| Pedosphaeraceae | 0.41 | 0.66 | 0.87 | 70.17 |

Table S4. Cecal microbial contributors to dissimilarity in treated and control mice. SIMPER analysis of the top twenty OTUs that contribute most to dissimilarity between PbOAC or SRM2710a treatment and control.

*PbOAc X Control*

| OTU | Taxonomy | PbOAc Average Abundance | Control Average Abundance | % Contribution | Cumulative Contribution |
| --- | --- | --- | --- | --- | --- |
| Otu000001 | *Akkermansia* | 9.1 | 12.31 | 0.8 | 0.8 |
| Otu000012 | Lachnospiraceae | 5.58 | 2.53 | 0.78 | 1.57 |
| Otu000034 | Lachnospiraceae | 4.63 | 1.73 | 0.73 | 2.3 |
| Otu000013 | Lachnospiraceae NK4A136 group | 3.32 | 5.03 | 0.66 | 2.96 |
| Otu000152 | unculture | 2.36 | 0 | 0.6 | 3.56 |
| Otu000038 | Ruminococcacea | 1.46 | 3.55 | 0.59 | 4.14 |
| Otu000052 | Lachnospiraceae NK4A136 group | 0.4 | 2.69 | 0.57 | 4.72 |
| Otu000002 | *Lactobacillus* | 10.26 | 8.11 | 0.54 | 5.26 |
| Otu000044 | *Bifidobacterium* | 3.09 | 2.83 | 0.53 | 5.79 |
| Otu000117 | Lachnospiraceae | 2.24 | 0.33 | 0.52 | 6.31 |
| Otu000020 | Lachnospiraceae | 4.18 | 5.73 | 0.51 | 6.82 |
| Otu000005 | *Bacteroides* | 4.65 | 5.95 | 0.47 | 7.29 |
| Otu000191 | Mollicutes *RF3* | 2.37 | 0.77 | 0.47 | 7.76 |
| Otu000004 | *Ruminococcus 2* | 5.96 | 5.47 | 0.47 | 8.23 |
| Otu000057 | Lachnospiraceae | 2.45 | 0.8 | 0.46 | 8.69 |
| Otu000137 | Mollicutes RF3 | 1.75 | 1.04 | 0.43 | 9.12 |
| Otu000040 | *Staphylococcus* | 4.21 | 2.55 | 0.42 | 9.55 |
| Otu000084 | Clostridiales vadinBB60 grou | 2.26 | 1.7 | 0.42 | 9.97 |
| Otu000101 | Ruminococcaceae UCG-014 | 1.6 | 1.66 | 0.42 | 10.38 |
| Otu000102 | Ruminococcaceae UCG-014 | 0.57 | 1.48 | 0.41 | 10.79 |

*SRM2710a X Control*

| OTU | Taxonomy | SRM2710a Average Abundance | Control Average Abundance | % Contribution | Cumulative Contribution |
| --- | --- | --- | --- | --- | --- |
| Otu000001 | *Akkermansia* | 9.25 | 12.31 | 0.78 | 0.78 |
| Otu000017 | *Clostridium sensu stricto 1* | 3.98 | 1.17 | 0.73 | 1.52 |
| Otu000005 | *Bacteroides* | 3.11 | 5.95 | 0.69 | 2.2 |
| Otu000044 | *Bifidobacterium* | 1.58 | 2.83 | 0.65 | 2.85 |
| Otu000091 | Lachnospiraceae | 3.42 | 1.11 | 0.62 | 3.47 |
| Otu000011 | *Romboutsia* | 3.31 | 1.11 | 0.56 | 4.04 |
| Otu000102 | Ruminococcaceae UCG-014 | 2.02 | 1.48 | 0.52 | 4.55 |
| Otu000051 | *Ruminococcus 2* | 2.31 | 1.13 | 0.51 | 5.06 |
| Otu000034 | Lachnospiraceae | 3.69 | 1.73 | 0.46 | 5.52 |
| Otu000137 | Mollicutes RF3 | 2.19 | 1.04 | 0.45 | 5.97 |
| Otu000021 | *Bacteroides* | 2.36 | 4.12 | 0.45 | 6.42 |
| Otu000014 | *Parabacteroides* | 2.53 | 4.38 | 0.44 | 6.87 |
| Otu000117 | Lachnospiraceae | 2.28 | 0.33 | 0.44 | 7.3 |
| Otu000012 | Lachnospiraceae | 4.33 | 2.53 | 0.44 | 7.74 |
| Otu000004 | *Ruminococcus 2* | 5.63 | 5.47 | 0.43 | 8.18 |
| Otu000002 | *Lactobacillus* | 8.85 | 8.11 | 0.43 | 8.61 |
| Otu000170 | Lachnospiraceae | 1.84 | 0 | 0.43 | 9.03 |
| Otu000054 | Lachnospiraceae NK4A136 group | 2.6 | 1.03 | 0.42 | 9.45 |
| Otu000048 | Lachnospiraceae NK4A136 group | 3.17 | 2.09 | 0.39 | 9.84 |
| Otu000023 | Ruminococcacea | 3.02 | 3.7 | 0.39 | 10.23 |

Table S5. Average sequences per genus per cecum sample in control and treatmented microbiomes. Each sample contained 41,931 sequences. Average similarity across all doses within a treatment, genus abundance, average percent similarity a genus contributes to within group similarity, average similarity over the standard deviation, and percent contributions to similarity. Average abundance is 4th root transformation of sequences in a genus.

| Control | | |  |  |
| --- | --- | --- | --- | --- |
| Average similarity: 72.79 |  |  |  |  |
| Species | Average Abundance | Average Similarity | Contribution % | Cumulative % |
| *Akkermansia* | 2.72 | 4.01 | 5.5 | 5.5 |
| *Lactobacillus* | 1.91 | 2.69 | 3.69 | 9.2 |
| Muribaculaceae_ge | 1.51 | 2.19 | 3 | 12.2 |
| Burkholderiaceae_unclassified | 1.63 | 2.16 | 2.96 | 15.16 |
| *Bacteroides* | 1.38 | 1.84 | 2.53 | 17.69 |
| *Bilophila* | 1.11 | 1.54 | 2.11 | 19.8 |
| uncultured | 0.98 | 1.44 | 1.98 | 21.79 |
| *Rhodanobacter* | 0.91 | 1.28 | 1.76 | 23.54 |
| *Sediminibacterium* | 0.86 | 1.27 | 1.74 | 25.28 |
| *Ruminococcus_2* | 0.9 | 1.26 | 1.74 | 27.02 |
| *Romboutsia* | 0.97 | 1.25 | 1.71 | 28.73 |
| *Polynucleobacter* | 0.86 | 1.23 | 1.69 | 30.43 |
| Lachnospiraceae_NK4A136_group | 0.94 | 1.23 | 1.69 | 32.11 |
| Rhodocyclaceae_unclassified | 0.87 | 1.14 | 1.57 | 33.68 |
| *Clostridium_sensu_stricto_1* | 0.91 | 1.08 | 1.48 | 35.16 |
| *Lachnoclostridium* | 0.78 | 1.05 | 1.44 | 36.6 |
| *Pseudomonas* | 0.75 | 1.02 | 1.4 | 38 |
| *Candidatus*_Aquiluna | 0.82 | 0.98 | 1.34 | 39.35 |
| Sphingomonadaceae_unclassified | 0.64 | 0.92 | 1.27 | 40.61 |
| *Arcobacter* | 0.68 | 0.91 | 1.25 | 41.86 |
| *Sulfurimonas* | 0.64 | 0.87 | 1.19 | 43.05 |
| *Nitrospira* | 0.61 | 0.86 | 1.19 | 44.24 |
| uncultured_ge | 0.6 | 0.86 | 1.19 | 45.43 |
| *Candidatus_Udaeobacter* | 0.61 | 0.85 | 1.17 | 46.6 |
| *Ruminococcus_1* | 0.59 | 0.84 | 1.16 | 47.76 |
| Ruminococcaceae_ge | 0.61 | 0.82 | 1.13 | 48.89 |
| Enterobacteriaceae_unclassified | 0.6 | 0.82 | 1.13 | 50.02 |
| *Candidatus_*Methylopumilus | 0.56 | 0.75 | 1.03 | 51.05 |
| *Pseudarcicella* | 0.5 | 0.73 | 1.01 | 52.05 |
| *C39* | 0.49 | 0.71 | 0.98 | 53.03 |
| Lachnospiraceae_unclassified | 0.54 | 0.67 | 0.92 | 53.95 |
| Ruminococcaceae_unclassified | 0.47 | 0.67 | 0.92 | 54.87 |
| Muribaculaceae_unclassified | 0.56 | 0.67 | 0.92 | 55.79 |
| *Rothia* | 0.47 | 0.67 | 0.92 | 56.7 |
| Sporichthyaceae_unclassified | 0.47 | 0.66 | 0.91 | 57.62 |
| hgcI_clade | 0.62 | 0.65 | 0.89 | 58.5 |
| *Candidatus_*Aquirestis | 0.48 | 0.63 | 0.86 | 59.37 |
| Planococcaceae_unclassified | 0.45 | 0.6 | 0.82 | 60.19 |
| *Terrimonas* | 0.46 | 0.59 | 0.82 | 61 |
| *Cyanobium_PCC-6307* | 0.43 | 0.58 | 0.79 | 61.8 |
| *Ruminiclostridium* | 0.45 | 0.56 | 0.77 | 62.57 |
| *Rhizorhapis* | 0.39 | 0.56 | 0.77 | 63.34 |
| Oxyphotobacteria_unclassified | 0.38 | 0.56 | 0.76 | 64.1 |
| *Rhodobacter* | 0.63 | 0.55 | 0.75 | 64.85 |
| *Burkholderia-Caballeronia-Paraburkholderia* | 0.5 | 0.54 | 0.74 | 65.59 |
| Pedosphaeraceae_ge | 0.41 | 0.54 | 0.74 | 66.33 |
| NS3a_marine_group | 0.38 | 0.53 | 0.73 | 67.06 |
| Beijerinckiaceae_unclassified | 0.4 | 0.53 | 0.73 | 67.79 |
| *Dechloromonas* | 0.43 | 0.52 | 0.72 | 68.5 |
| Ruminococcaceae_UCG-005 | 0.4 | 0.52 | 0.71 | 69.21 |
| *Bifidobacterium* | 0.48 | 0.51 | 0.7 | 69.92 |
| *Enterococcus* | 0.39 | 0.51 | 0.7 | 70.62 |
|  |  |  |  |  |
| *PbOAc* | |  |  |  |
| Average similarity: 66.12 |  |  |  |  |
| Species | Average Abundance | Average Similarity | Contribution % | Cumulative % |
| *Lactobacillus* | 2.32 | 3.04 | 4.6 | 4.6 |
| *Akkermansia* | 2.01 | 2.47 | 3.73 | 8.33 |
| Muribaculaceae_ge | 1.61 | 2.08 | 3.15 | 11.48 |
| Burkholderiaceae_unclassified | 1.62 | 2.02 | 3.05 | 14.54 |
| *Bacteroides* | 1.45 | 1.72 | 2.59 | 17.13 |
| uncultured | 1.07 | 1.41 | 2.13 | 19.26 |
| *Rhodanobacter* | 1.1 | 1.38 | 2.09 | 21.34 |
| Rhodocyclaceae_unclassified | 1.12 | 1.33 | 2.02 | 23.36 |
| Muribaculaceae_unclassified | 1.23 | 1.27 | 1.93 | 25.29 |
| Lachnospiraceae_NK4A136_group | 0.85 | 1.07 | 1.62 | 26.91 |
| *Mucispirillum* | 1.02 | 1.05 | 1.59 | 28.5 |
| *Sediminibacterium* | 0.81 | 1.03 | 1.56 | 30.06 |
| Ruminococcaceae_ge | 0.88 | 1.01 | 1.53 | 31.59 |
| *Arcobacter* | 0.84 | 1.01 | 1.52 | 33.11 |
| *Pseudomonas* | 0.86 | 0.97 | 1.47 | 34.59 |
| *Romboutsia* | 0.82 | 0.92 | 1.4 | 35.98 |
| *Candidatus_*Aquiluna | 0.78 | 0.89 | 1.35 | 37.33 |
| Lachnospiraceae_unclassified | 0.77 | 0.86 | 1.31 | 38.63 |
| *Polynucleobacter* | 0.71 | 0.85 | 1.28 | 39.91 |
| uncultured_ge | 0.71 | 0.84 | 1.27 | 41.19 |
| *Candidatus_*Udaeobacter | 0.64 | 0.81 | 1.23 | 42.41 |
| *Cyanobium_PCC-6307* | 0.63 | 0.81 | 1.22 | 43.64 |
| *Clostridium_sensu_stricto_1* | 0.75 | 0.79 | 1.2 | 44.84 |
| *Ruminococcus_2* | 0.71 | 0.79 | 1.2 | 46.03 |
| *Pseudarcicella* | 0.68 | 0.79 | 1.2 | 47.23 |
| *Nitrospira* | 0.59 | 0.74 | 1.13 | 48.36 |
| *Candidatus_*Methylopumilus | 0.55 | 0.68 | 1.04 | 49.39 |
| Sphingomonadaceae_unclassified | 0.54 | 0.65 | 0.98 | 50.37 |
| *Ruminococcus_1* | 0.53 | 0.64 | 0.97 | 51.34 |
| *Ruminiclostridium* | 0.51 | 0.63 | 0.95 | 52.29 |
| Pedosphaeraceae_ge | 0.54 | 0.6 | 0.91 | 53.2 |
| *Sulfurimonas* | 0.52 | 0.57 | 0.87 | 54.07 |
| *Bryobacter* | 0.49 | 0.56 | 0.85 | 54.91 |
| Ruminococcaceae_UCG-005 | 0.46 | 0.55 | 0.84 | 55.75 |
| Sporichthyaceae_unclassified | 0.44 | 0.55 | 0.84 | 56.58 |
| *Bilophila* | 0.73 | 0.55 | 0.83 | 57.42 |
| *Rhodobacter* | 0.68 | 0.55 | 0.83 | 58.25 |
| *Rothia* | 0.49 | 0.55 | 0.83 | 59.07 |
| hgcI_clade | 0.47 | 0.54 | 0.81 | 59.89 |
| Planococcaceae_unclassified | 0.44 | 0.54 | 0.81 | 60.7 |
| Ruminococcaceae_UCG-014 | 0.43 | 0.53 | 0.8 | 61.5 |
| Ruminococcaceae_unclassified | 0.46 | 0.52 | 0.79 | 62.29 |
| *Candidatus_*Aquirestis | 0.45 | 0.51 | 0.77 | 63.06 |
| *Erysipelatoclostridium* | 0.54 | 0.51 | 0.77 | 63.83 |
| C39 | 0.43 | 0.5 | 0.75 | 64.58 |
| SC-I-84_ge | 0.4 | 0.49 | 0.74 | 65.32 |
| Enterobacteriaceae_unclassified | 0.42 | 0.48 | 0.73 | 66.05 |
| *Sulfurifustis* | 0.43 | 0.47 | 0.72 | 66.77 |
| *Rhizorhapis* | 0.41 | 0.46 | 0.7 | 67.47 |
| *Shimwellia* | 0.37 | 0.45 | 0.68 | 68.15 |
| Methylomonaceae_unclassified | 0.39 | 0.44 | 0.67 | 68.81 |
| NS11-12_marine_group_ge | 0.38 | 0.42 | 0.63 | 69.44 |
| *Porphyrobacter* | 0.39 | 0.41 | 0.62 | 70.07 |
|  |  |  |  |  |
| *Group 2710a* |  |  |  |  |
| Average similarity: 64.49 |  |  |  |  |
| Species | Average Abundance | Average Similarity | Contribution % | Cumulative % |
| *Lactobacillus* | 2.25 | 2.66 | 4.12 | 4.12 |
| *Akkermansia* | 2.04 | 2.28 | 3.53 | 7.65 |
| *Bilophila* | 1.37 | 1.74 | 2.7 | 10.35 |
| Burkholderiaceae_unclassified | 1.43 | 1.6 | 2.47 | 12.82 |
| Muribaculaceae_ge | 1.31 | 1.47 | 2.28 | 15.1 |
| *Bacteroides* | 1.27 | 1.45 | 2.25 | 17.35 |
| uncultured | 1.11 | 1.35 | 2.09 | 19.44 |
| Lachnospiraceae_NK4A136_group | 1.25 | 1.19 | 1.85 | 21.28 |
| *Rhodanobacter* | 0.97 | 1.12 | 1.74 | 23.02 |
| Muribaculaceae_unclassified | 0.96 | 1.11 | 1.72 | 24.74 |
| Rhodocyclaceae_unclassified | 0.89 | 1.05 | 1.62 | 26.37 |
| Enterobacteriaceae_unclassified | 0.91 | 0.98 | 1.52 | 27.88 |
| *Sediminibacterium* | 0.83 | 0.94 | 1.46 | 29.34 |
| *Mucispirillum* | 0.82 | 0.91 | 1.42 | 30.76 |
| *Roseburia* | 0.84 | 0.89 | 1.39 | 32.15 |
| Lachnospiraceae_unclassified | 0.74 | 0.89 | 1.38 | 33.52 |
| Ruminococcaceae_ge | 0.84 | 0.82 | 1.27 | 34.8 |
| *Bifidobacterium* | 0.76 | 0.78 | 1.22 | 36.01 |
| *Pseudomonas* | 0.65 | 0.73 | 1.14 | 37.15 |
| *Pseudarcicella* | 0.67 | 0.73 | 1.13 | 38.28 |
| *Arcobacter* | 0.66 | 0.72 | 1.11 | 39.4 |
| *Polynucleobacter* | 0.64 | 0.72 | 1.11 | 40.51 |
| *Candidatus_*Methylopumilus | 0.63 | 0.68 | 1.05 | 41.56 |
| *Cyanobium_PCC-6307* | 0.6 | 0.68 | 1.05 | 42.61 |
| *Nitrospira* | 0.59 | 0.67 | 1.04 | 43.66 |
| *Bryobacter* | 0.59 | 0.66 | 1.03 | 44.68 |
| *Ruminococcus_1* | 0.56 | 0.66 | 1.03 | 45.71 |
| *Sulfurimonas* | 0.55 | 0.63 | 0.98 | 46.69 |
| hgcI_clade | 0.65 | 0.63 | 0.97 | 47.66 |
| Sphingomonadaceae_unclassified | 0.54 | 0.62 | 0.97 | 48.63 |
| *Romboutsia* | 0.56 | 0.6 | 0.94 | 49.56 |
| Ruminococcaceae_UCG-005 | 0.53 | 0.6 | 0.93 | 50.49 |
| *Candidatus_*Aquirestis | 0.51 | 0.59 | 0.92 | 51.42 |
| *Terrimonas* | 0.51 | 0.58 | 0.91 | 52.32 |
| uncultured_ge | 0.54 | 0.58 | 0.9 | 53.23 |
| *Candidatus_*Aquiluna | 0.67 | 0.57 | 0.89 | 54.12 |
| *Candidatus_*Udaeobacter | 0.52 | 0.57 | 0.88 | 55 |
| *Ruminococcus_2* | 0.56 | 0.57 | 0.88 | 55.88 |
| *Clostridium_sensu_stricto_1* | 0.55 | 0.57 | 0.88 | 56.76 |
| Clade_III_ge | 0.5 | 0.55 | 0.86 | 57.62 |
| *Rothia* | 0.5 | 0.54 | 0.84 | 58.46 |
| Pedosphaeraceae_ge | 0.5 | 0.54 | 0.83 | 59.29 |
| *Erysipelatoclostridium* | 0.52 | 0.53 | 0.82 | 60.11 |
| Sporichthyaceae_unclassified | 0.44 | 0.51 | 0.79 | 60.9 |
| *Crenothrix* | 0.43 | 0.5 | 0.77 | 61.67 |
| Planococcaceae_unclassified | 0.43 | 0.49 | 0.77 | 62.43 |
| CL500-29_marine_group | 0.46 | 0.47 | 0.73 | 63.16 |
| FukuN18_freshwater_group | 0.4 | 0.46 | 0.71 | 63.87 |
| Gammaproteobacteria_unclassified | 0.41 | 0.45 | 0.69 | 64.56 |
| Ruminococcaceae_UCG-014 | 0.38 | 0.44 | 0.69 | 65.25 |
| *Oscillibacter* | 0.39 | 0.44 | 0.68 | 65.94 |
| *Sulfuricurvum* | 0.63 | 0.44 | 0.68 | 66.61 |
| *Ruminiclostridium* | 0.47 | 0.43 | 0.67 | 67.29 |
| NS11-12_marine_group_ge | 0.4 | 0.42 | 0.66 | 67.95 |
| *Porphyrobacter* | 0.42 | 0.42 | 0.66 | 68.6 |
| C39 | 0.41 | 0.42 | 0.66 | 69.26 |
| *Lachnoclostridium* | 0.52 | 0.42 | 0.65 | 69.91 |
| *Dechloromonas* | 0.48 | 0.41 | 0.64 | 70.55 |

Table S6. Average sequences per family per cecum sample in control and treated microbiomes. Each sample contained 41,931 sequences. Average similarity across all doses within a treatment, family abundance, average percent similarity a family contributes to within group similarity, average similarity over the standard deviation, and percent contributions to similarity. Average abundance is 4th root transformation of sequences in a family.

| *Control* | | |  |  |
| --- | --- | --- | --- | --- |
| Average similarity: 76.79 |  |  |  |  |
| Family | Average Abundance | Average Similarity | Contribution % | Cumulative % |
| Akkermansiaceae | 2.72 | 4.97 | 6.47 | 6.47 |
| Lactobacillaceae | 1.91 | 3.34 | 4.34 | 10.82 |
| Burkholderiaceae | 1.67 | 2.76 | 3.59 | 14.41 |
| Muribaculaceae | 1.52 | 2.75 | 3.58 | 17.99 |
| Bacteroidaceae | 1.38 | 2.28 | 2.97 | 20.96 |
| Desulfovibrionaceae | 1.11 | 1.91 | 2.48 | 23.45 |
| Ruminococcaceae | 1.04 | 1.86 | 2.42 | 25.87 |
| Lachnospiraceae | 1.07 | 1.74 | 2.27 | 28.14 |
| Chitinophagaceae | 0.92 | 1.69 | 2.21 | 30.35 |
| Rhodanobacteraceae | 0.91 | 1.59 | 2.07 | 32.41 |
| Peptostreptococcaceae | 0.97 | 1.55 | 2.01 | 34.43 |
| Rhodocyclaceae | 0.91 | 1.5 | 1.95 | 36.38 |
| Peptococcaceae | 0.84 | 1.48 | 1.93 | 38.31 |
| Clostridiaceae_1 | 0.91 | 1.34 | 1.74 | 40.05 |
| Pseudomonadaceae | 0.75 | 1.27 | 1.65 | 41.7 |
| Sphingomonadaceae | 0.68 | 1.23 | 1.6 | 43.3 |
| Microbacteriaceae | 0.82 | 1.22 | 1.59 | 44.89 |
| Arcobacteraceae | 0.68 | 1.13 | 1.47 | 46.36 |
| Rhizobiales_Incertae_Sedis | 0.6 | 1.09 | 1.42 | 47.77 |
| Thiovulaceae | 0.65 | 1.09 | 1.41 | 49.19 |
| Chthoniobacteraceae | 0.62 | 1.08 | 1.41 | 50.6 |
| Nitrospiraceae | 0.61 | 1.07 | 1.4 | 51.99 |
| uncultured | 0.59 | 1.06 | 1.38 | 53.37 |
| Enterobacteriaceae | 0.6 | 1.03 | 1.34 | 54.71 |
| Sporichthyaceae | 0.7 | 1.02 | 1.32 | 56.03 |
| Methylophilaceae | 0.56 | 0.94 | 1.22 | 57.25 |
| Spirosomaceae | 0.51 | 0.92 | 1.2 | 58.45 |
| Steroidobacteraceae | 0.53 | 0.9 | 1.17 | 59.62 |
| Micrococcaceae | 0.47 | 0.83 | 1.08 | 60.7 |
| Saprospiraceae | 0.48 | 0.79 | 1.03 | 61.73 |
| Methylomonaceae | 0.46 | 0.79 | 1.02 | 62.75 |
| Rhodobacteraceae | 0.65 | 0.76 | 0.99 | 63.74 |
| Planococcaceae | 0.45 | 0.74 | 0.97 | 64.71 |
| Cyanobiaceae | 0.43 | 0.72 | 0.93 | 65.64 |
| Ilumatobacteraceae | 0.41 | 0.72 | 0.93 | 66.57 |
| Moraxellaceae | 0.44 | 0.71 | 0.92 | 67.5 |
| Beijerinckiaceae | 0.42 | 0.69 | 0.9 | 68.4 |
| Oxyphotobacteria_unclassified | 0.38 | 0.69 | 0.9 | 69.3 |
| Pedosphaeraceae | 0.41 | 0.66 | 0.87 | 70.17 |
|  |  |  |  |  |
| *PbOAc* |  |  |  |  |
| Average similarity: 69.76 |  |  |  |  |
| Family | Average Abundance | Average Similarity | Contribution % | Cumulative % |
| Lactobacillaceae | 2.32 | 3.72 | 5.33 | 5.33 |
| Akkermansiaceae | 2.01 | 3.01 | 4.32 | 9.65 |
| Muribaculaceae | 1.82 | 2.8 | 4.01 | 13.66 |
| Burkholderiaceae | 1.66 | 2.52 | 3.62 | 17.28 |
| Bacteroidaceae | 1.45 | 2.1 | 3.01 | 20.29 |
| Lachnospiraceae | 1.11 | 1.71 | 2.45 | 22.74 |
| Rhodanobacteraceae | 1.1 | 1.69 | 2.42 | 25.16 |
| Rhodocyclaceae | 1.14 | 1.68 | 2.41 | 27.58 |
| Ruminococcaceae | 1.1 | 1.66 | 2.39 | 29.96 |
| Chitinophagaceae | 0.9 | 1.4 | 2 | 31.97 |
| Deferribacteraceae | 1.02 | 1.29 | 1.85 | 33.82 |
| Arcobacteraceae | 0.84 | 1.24 | 1.77 | 35.59 |
| Pseudomonadaceae | 0.86 | 1.19 | 1.71 | 37.3 |
| Peptostreptococcaceae | 0.82 | 1.13 | 1.62 | 38.92 |
| Microbacteriaceae | 0.78 | 1.09 | 1.57 | 40.48 |
| Rhizobiales_Incertae_Sedis | 0.7 | 1.07 | 1.53 | 42.01 |
| Sphingomonadaceae | 0.67 | 1.01 | 1.44 | 43.45 |
| Chthoniobacteraceae | 0.64 | 1 | 1.43 | 44.88 |
| Cyanobiaceae | 0.63 | 0.99 | 1.42 | 46.3 |
| Clostridiaceae_1 | 0.75 | 0.98 | 1.4 | 47.7 |
| Spirosomaceae | 0.68 | 0.97 | 1.4 | 49.1 |
| Peptococcaceae | 0.68 | 0.91 | 1.31 | 50.41 |
| Nitrospiraceae | 0.59 | 0.91 | 1.31 | 51.71 |
| uncultured | 0.66 | 0.89 | 1.28 | 52.99 |
| Sporichthyaceae | 0.57 | 0.88 | 1.27 | 54.26 |
| Methylophilaceae | 0.58 | 0.87 | 1.24 | 55.51 |
| Erysipelotrichaceae | 0.61 | 0.79 | 1.13 | 56.64 |
| Enterobacteriaceae | 0.51 | 0.78 | 1.12 | 57.76 |
| Pedosphaeraceae | 0.54 | 0.74 | 1.07 | 58.83 |
| Rhodobacteraceae | 0.71 | 0.73 | 1.05 | 59.88 |
| Methylomonaceae | 0.49 | 0.72 | 1.04 | 60.92 |
| Steroidobacteraceae | 0.48 | 0.72 | 1.03 | 61.95 |
| Thiovulaceae | 0.52 | 0.72 | 1.03 | 62.97 |
| Solibacteraceae_ | 0.5 | 0.7 | 1 | 63.97 |
| Desulfovibrionaceae | 0.73 | 0.68 | 0.98 | 64.95 |
| Micrococcaceae | 0.49 | 0.67 | 0.96 | 65.91 |
| Ilumatobacteraceae | 0.48 | 0.66 | 0.94 | 66.85 |
| Planococcaceae | 0.44 | 0.66 | 0.94 | 67.79 |
| Saprospiraceae | 0.46 | 0.66 | 0.94 | 68.73 |
| SC-I-84 | 0.4 | 0.6 | 0.86 | 69.6 |
| Acidiferrobacteraceae | 0.43 | 0.58 | 0.82 | 70.42 |
|  |  |  |  |  |
| *SRM 2710a* |  |  |  |  |
| Average similarity: 68.63 |  |  |  |  |
| Family | Average Abundance | Average Similarity | Contribution % | Cumulative % |
| Lactobacillaceae | 2.25 | 3.32 | 4.84 | 4.84 |
| Akkermansiaceae | 2.04 | 2.84 | 4.14 | 8.98 |
| Desulfovibrionaceae | 1.37 | 2.17 | 3.17 | 12.15 |
| Muribaculaceae | 1.42 | 2.08 | 3.03 | 15.18 |
| Burkholderiaceae | 1.45 | 2.04 | 2.97 | 18.15 |
| Lachnospiraceae | 1.49 | 1.98 | 2.88 | 21.03 |
| Bacteroidaceae | 1.27 | 1.81 | 2.64 | 23.67 |
| Rhodocyclaceae | 0.96 | 1.43 | 2.09 | 25.76 |
| Ruminococcaceae | 1.04 | 1.43 | 2.09 | 27.85 |
| Rhodanobacteraceae | 0.97 | 1.4 | 2.05 | 29.89 |
| Chitinophagaceae | 0.93 | 1.34 | 1.95 | 31.85 |
| Enterobacteriaceae | 0.93 | 1.26 | 1.83 | 33.68 |
| Peptococcaceae | 0.85 | 1.18 | 1.72 | 35.4 |
| Deferribacteraceae | 0.82 | 1.14 | 1.67 | 37.07 |
| Bifidobacteriaceae | 0.76 | 0.98 | 1.43 | 38.5 |
| Spirosomaceae | 0.69 | 0.95 | 1.38 | 39.88 |
| Rhizobiales_Incertae_Sedis | 0.67 | 0.94 | 1.37 | 41.25 |
| Sphingomonadaceae | 0.67 | 0.93 | 1.36 | 42.61 |
| Pseudomonadaceae | 0.65 | 0.92 | 1.34 | 43.94 |
| Sporichthyaceae | 0.7 | 0.91 | 1.33 | 45.27 |
| Arcobacteraceae | 0.66 | 0.9 | 1.31 | 46.58 |
| Thiovulaceae | 0.8 | 0.89 | 1.29 | 47.87 |
| Solibacteraceae_ | 0.6 | 0.86 | 1.25 | 49.12 |
| Methylophilaceae | 0.63 | 0.85 | 1.24 | 50.35 |
| Cyanobiaceae | 0.61 | 0.85 | 1.23 | 51.59 |
| Nitrospiraceae | 0.59 | 0.84 | 1.22 | 52.81 |
| Microbacteriaceae | 0.7 | 0.83 | 1.2 | 54.01 |
| Steroidobacteraceae | 0.56 | 0.77 | 1.12 | 55.13 |
| Saprospiraceae | 0.52 | 0.76 | 1.11 | 56.24 |
| Peptostreptococcaceae | 0.56 | 0.76 | 1.1 | 57.34 |
| Erysipelotrichaceae | 0.57 | 0.75 | 1.09 | 58.43 |
| Chthoniobacteraceae | 0.53 | 0.75 | 1.09 | 59.52 |
| Methylomonaceae | 0.5 | 0.73 | 1.06 | 60.58 |

Table S7. Predicted functional gene average abundance in cecal microbiome from PbOAc and SRM 2710a treated mice and Pb-free control. The 16S sequences from the top 20 OTUs contributing most to dissimilarity at the highest dose of PbOAc (25 mg/kg) and SRM 2710a (30 mg/kg) were analyzed using the CLC Genomics Workbench (22.0.2; Microbial Genomics Module) as described. MetaCyc (metacyc.org) (Caspi et al., 2020) and Transform Column-All Ancestors of Entity were used to identify super pathways.

| **MetaCyc ID** | **Functional Name** | **PbOAc** | **SRM 2710a** | **Control** |
| --- | --- | --- | --- | --- |
|  |  | **Average Gene Abundance** | **Average Gene Abundance** | **Average Gene Abundance** |
| **Metal Resistance** |  |  |  |  |
| AEROBACTINSYN-PWY | aerobactin biosynthesis (siderophore) | 1,082.00 | 144 | 7 |
| PWY-6421 | arsenate detoxification IV (mycothiol) (As) | 52 | 217 | 0 |
| P641-PWY | phenylmercury acetate degradation (Hg) | 1 | 431 | 14 |
|  |  |  |  |  |
| **Antibiotic Resistance** |  |  |  |  |
| PWY-6454 | vancomycin resistance I | 19,983.67 | 13,962.67 | 22,588.33 |
| PWY-6455 | vancomycin resistance II | 11,791.67 | 7,333.33 | 11,544.67 |
| PWY0-1338 | polymyxin resistance | 310.4 | 927.4 | 37.8 |
| PWY-6679 | jadomycin biosynthesis | 58,180.50 | 89,270.50 | 31,766.50 |
| PWY-5770 | phenazine-1-carboxylate biosynthesis | 1,649.00 | 2,931.00 | 578 |
| PWY-7485 | tetracenomycin C biosynthesis | 176.25 | 464.5 | 756 |
|  |  |  |  |  |
| **Carbohydrates Biosynthesis** |  |  |  |  |
| ECASYN-PWY | enterobacterial common antigen biosynthesis | 29,239.29 | 28,766.57 | 31,434.29 |
| PWY-7905 | Escherichia coli serotype O9a O-antigen biosynthesis | 12,497.00 | 10,405.33 | 5,980.67 |
| PWY-822 | fructan biosynthesis | 220 | 284 | 404 |
| GLUCONEO-PWY | gluconeogenesis I | 53,016.46 | 72,956.31 | 44,543.15 |
| PWY-8013 | UDP-N-acetyl-D-galactosamine biosynthesis III | 34,470.67 | 45,617.33 | 27,948.67 |
| PWY-4821 | UDP-&alpha;-D-xylose biosynthesis | 24,455.00 | 58,854.50 | 34,105.00 |
| PWY-1269 | CMP-3-deoxy-D- manno -octulosonate biosynthesis | 17,601.75 | 6,686.00 | 24,874.50 |
| PWY0-1241 | ADP-L-glycero-&beta;-D-manno-heptose biosynthesis | 16,872.60 | 7,650.40 | 20,055.40 |
| PWY-6478 | GDP-D-glycero-&alpha;-D-manno-heptose biosynthesis | 14,051.25 | 4,707.00 | 13,633.50 |
| PWY-7131 | CMP-legionaminate biosynthesis II | 13,753.67 | 6,139.00 | 5,029.00 |
| PWY-6143 | CMP-pseudaminate biosynthesis | 2,128.67 | 371.33 | 322.17 |
| PWY-7028 | UDP-N,N'-diacetylbacillosamine biosynthesis | 1,540.00 | 217 | 185 |
|  | SUM | 219,846.36 | 242,654.77 | 208,515.35 |
| **Lipid Biosynthesis** |  |  |  |  |
| PWY0-1264 | biotin-carboxyl carrier protein assembly | 45,006.00 | 64,226.00 | 34,707.00 |
| PWYQT-4427 | sulfoquinovosyl diacylglycerol biosynthesis | 3,831.00 | 5,264.00 | 2,197.00 |
| PWY0-541 | cyclopropane fatty acid (CFA) biosynthesis | 50,945.00 | 25,909.00 | 20,310.00 |
| PWY-7902 | glucosylglycerol biosynthesis | 32,991.60 | 66,692.80 | 24,879.40 |
| PWY-7509 | cardiolipin and phosphatidylethanolamine biosynthesis | 18,563.00 | 10,577.00 | 24,307.67 |
| PWY-6350 | archaetidylinositol biosynthesis | 549.33 | 3,435.43 | 369.29 |
|  | SUM | 151,885.93 | 176,104.23 | 106,770.36 |
| **Carbohydrate Degradation** |  |  |  |  |
| PWY-7586 | &beta;-1,4-D-mannosyl-N-acetyl-D-glucosamine degradation | 30,767.00 | 43,130.33 | 23,996.67 |
| RIBOKIN-PWY | ribose phosphorylation | 49,001.00 | 103,221.00 | 24,940.50 |
| PWY0-1301 | melibiose degradation | 47,102.00 | 26,693.00 | 70,623.00 |
| PWY0-1309 | chitobiose degradation | 40,448.50 | 137,561.00 | 16,888.00 |
| PWY0-1314 | fructose degradation | 25,653.00 | 60,900.00 | 15,365.00 |
| MALTOSECAT-PWY | maltose degradation | 17,488.50 | 78,295.00 | 12,186.50 |
| PWY-7459 | kojibiose degradation | 8,465.50 | 55,486.00 | 5,009.00 |
| PWY-8121 | 2-deoxy-D-glucose 6-phosphate degradation | 2,364.00 | 36,949.00 | 2,326.00 |
| PWY0-44 | D-allose degradation | 120 | 618 | 756 |
| LACTOSEUTIL-PWY | lactose degradation II | 146,475.00 | 129,649.00 | 246,348.00 |
| P124-PWY | Bifidobacterium shunt | 62,528.53 | 91,495.33 | 45,122.60 |
| PWY-621 | sucrose degradation III (sucrose invertase) | 61,088.50 | 80,071.75 | 50,334.75 |
| PWY-6317 | D-galactose degradation I (Leloir pathway) | 53,009.60 | 92,697.60 | 54,558.00 |
| PWY-5384 | sucrose degradation IV (sucrose phosphorylase) | 46,411.75 | 80,686.75 | 44,187.25 |
| TREDEGLOW-PWY | trehalose degradation I (low osmolarity) | 40,471.50 | 57,488.00 | 34,834.00 |
| PWY-3801 | sucrose degradation II (sucrose synthase) | 39,605.40 | 94,785.60 | 40,162.20 |
| PWY-2722 | trehalose degradation IV | 26,050.33 | 102,437.00 | 23,447.00 |
| PWY-7581 | N-acetylneuraminate and N-acetylmannosamine degradation II | 20,426.50 | 30,103.50 | 33,873.00 |
| PWY-8058 | 2-deoxy-D-ribose degradation II | 19,533.00 | 69,060.33 | 12,457.67 |
| LACTOSECAT-PWY | lactose and galactose degradation I | 8,070.50 | 29,553.50 | 3,732.75 |
| GALDEG-PWY | D-galactose degradation II | 220 | 284 | 404 |
| PWY-6784 | cellulose and hemicellulose degradation (cellulolosome) | 11,111.00 | 2,930.00 | 3,507.50 |
| GLYCOCAT-PWY | glycogen degradation I | 51,287.00 | 85,023.29 | 50,624.57 |
| PWY-6906 | chitin derivatives degradation | 37,876.75 | 38,145.00 | 32,477.00 |
| PWY-7644 | heparin degradation | 25,316.67 | 5,798.67 | 7,119.67 |
| PWY-7456 | &beta;-(1,4)-mannan degradation | 20,424.60 | 26,260.80 | 19,029.20 |
| PWY-6986 | alginate degradation | 19,141.50 | 43,679.00 | 10,041.00 |
| PWY-6717 | (1,4)-&beta;-D-xylan degradation | 11,169.00 | 18,829.00 | 3,310.00 |
| PWY-7651 | heparan sulfate degradation | 11,138.50 | 2,502.00 | 3,428.50 |
| PWY-6572 | chondroitin sulfate degradation I (bacterial) | 2,156.33 | 11,096.00 | 4,449.33 |
| PWY-6771 | rhamnogalacturonan type I degradation II (bacteria) | 408 | 303 | 11 |
| PWY-7645 | hyaluronan degradation | 280 | 32,366.00 | 386.5 |
|  | SUM | 935,609.46 | 1,668,098.45 | 895,936.16 |
| **Fatty Acid & Lipid Degradation** |  |  |  |  |
| LIPASYN-PWY | phospholipases | 4,161.67 | 26,466.33 | 4,246.33 |
| LIPAS-PWY | triacylglycerol degradation | 5,148.00 | 2,256.00 | 1,233.00 |
| PWY-5533 | acetone degradation II (to acetoacetate) | 1 | 646 | 20 |
| PWY0-1337 | oleate &beta;-oxidation | 7,939.88 | 33,275.88 | 5,828.72 |
| FAO-PWY | fatty acid &beta;-oxidation I (generic) | 2,684.40 | 16,669.20 | 1,864.60 |
| PWY-8135 | bile acids deconjugation | 64,943.00 | 34,381.00 | 37,670.00 |
|  | SUM | 84,877.95 | 113,694.41 | 50,862.65 |

Fig. S1. Relative abundances of cecal Classes. Values are mean numbers of sequences across treatment doses with error bars being the standard error of the mean. Control diet class relative abundances are in rank order.

**References**

Caspi, R., Billington, R., Keseler, I. M., Kothari, A., Krummenacker, M., Midford, P. E., . . . Karp, P. D. (2020). The MetaCyc database of metabolic pathways and enzymes-a 2019 update. *Nucleic acids research, 48*(D1), D445-D453.
